# A pangenome analysis reveals the center of origin and evolutionary history of *Phytophthora infestans* and 1c clade species

**DOI:** 10.1371/journal.pone.0314509

**Published:** 2025-01-24

**Authors:** Allison L. Coomber, Amanda C. Saville, Ignazio Carbone, Michael Martin, Vanessa C. Bieker, Jean Beagle Ristaino

**Affiliations:** 1 Department of Entomology and Plant Pathology, NC State University, Raleigh, North Carolina, United States of America; 2 Functional Genomics Program, NC State University, Raleigh, North Carolina, United States of America; 3 Center for Integrated Fungal Research, North Carolina State University, Raleigh, NC, United States of America; 4 Department of Natural History, NTNU University Museum, Norwegian University of Science and Technology (NTNU), Trondheim, Norway; 5 Emerging Plant Disease and Global Food Security Cluster, NC State University, Raleigh, North Carolina, United States of America; Franklin & Marshall College, UNITED STATES OF AMERICA

## Abstract

We examined the evolutionary history of *Phytophthora infestans* and its close relatives in the 1c clade. We used whole genome sequence data from 69 isolates of *Phytophthora* species in the 1c clade and conducted a range of genomic analyses including nucleotide diversity evaluation, maximum likelihood trees, network assessment, time to most recent common ancestor and migration analysis. We consistently identified distinct and later divergence of the two Mexican *Phytophthora* species, *P*. *mirabilis* and *P*. *ipomoeae*, from *P*. *infestans* and other 1c clade species. *Phytophthora infestans* exhibited more recent divergence from other 1c clade species of *Phytophthora* from South America, *P*. *andina* and *P*. *betacei*. Speciation in the 1c clade and evolution of *P*. *infestans* occurred in the Andes. *P*. *andina–P*. *betacei–P*. *infestans* formed a species complex with indistinct species boundaries, hybridizations between the species, and short times to common ancestry. Furthermore, the distinction between modern Mexican and South American *P*. *infestans* proved less discrete, suggesting gene flow between populations over time. Admixture analysis indicated a complex relationship among these populations, hinting at potential gene flow across these regions. Historic *P*. *infestans*, collected from 1845–1889, were the first to diverge from all other *P*. *infestans* populations. Modern South American populations diverged next followed by Mexican populations which showed later ancestry. Both populations were derived from historic *P*. *infestans*. Based on the time of divergence of *P*. *infestans* from its closest relatives, *P*. *andina* and *P*. *betacei* in the Andean region, we consider the Andes to be the center of origin of *P*. *infestans*, with modern globalization contributing to admixture between *P*. *infestans* populations today from Mexico, the Andes and Europe.

## Introduction

*Phytophthora infestans* is a plant-pathogenic oomycete that infects potatoes, tomatoes, and their close Solanaceous relatives [1]. The pathogen caused the Irish Potato Famine between 1845–1852 and has been the subject of extensive study since the inception of the field of plant pathology [2]. The historical significance of this pathogen continues to captivate scientists to this day [1]. Studies have identified and tracked the historic lineages responsible for the Irish Potato Famine outbreaks using DNA from herbarium specimens [3]. The pathogen has been disseminated globally and ancestral mitochondrial haplotype (HERB-1) and the famine lineage (FAM-1) of *P*. *infestans* have been identified and were found to persist for over 100 years after the 1840s outbreaks [3–5]. Researchers have proposed various hypotheses about the pathogen’s origin, including a Mexican origin, an Andean origin, and a hybrid origin theory. Investigations have been conducted to ascertain both the origin and migration patterns of the pathogen into the US and Europe. However, a consensus regarding the precise center of origin of *P*. *infestans* and the entire 1c clade of related sister species remains controversial [3–5].

*Phytophthora infestans* is a member of a genus encompassing over 200 species [6–8]. Traditionally, this genus has been divided into clades, with *P*. *infestans* falling into clade 1c [6, 7, 9, 10]. This particular subclade includes six additional species: *P*. *phaseoli*, a pathogen affecting the lima bean (*Phaseolus lunatus*); *P*. *mirabilis*, a pathogen of the Peruvian 4 o’clock flower (*Mirabilis jalapa*); *P*. *ipomoeae*, a pathogen of Mexican *Ipomoea* species; *P*. *betacei*, a recently described pathogen of tree tomatoes (*Solanum betaceum*) in Colombia; *P*. *andina*, a hybrid between *P*. *infestans* and an as-yet unidentified lineage in clade 1c; and the most recently reported *P*. *urerae*, a pathogen that infects *Urera laciniata*, a host found in the Peruvian Andes [11–18]. Some but not all of these closely related clade 1c species have been included in previous evolution and origin studies [16, 19].

The discussion surrounding the origin of *P*. *infestans* is steeped in history. Presently, the theories of a South American or Mexican origin stand as the primary contenders in the ongoing debate [19–22]. In 2002, Ristaino assessed the evidence for both the Mexican and South American origin hypotheses [3]. She pointed to the absence of potato exports during the 1840s, which posed a challenge to the notion of a Mexican origin for the blight’s migration to the US and Europe [3]. Furthermore, historical accounts of a similar disease in the Andean region and the presence of the cosmopolitan US-1 lineage in South America since at least the 1980s (yet absent in Mexico) were invoked by Ristaino, potentially supporting the idea of a South American origin [3].

In 2005, Grünwald and Flier issued a review titled “The Biology of *Phytophthora infestans* at Its Center of Origin,” asserting Mexico as the center of origin based on sexual reproduction of the pathogen there and population genetics studies using mitochondrial haplotyping, AFLP fingerprinting, and other multilocus marker studies conducted by the same research group [16]. Their proposed phylogeny of the 1c clade *Phytophthora* species involved host switching events preceding speciation [16]. However, in 2006, the Luis Gomez-Alpizar from the Ristaino lab (Gómez-Alpizar et al. [19]) challenged these conclusions with additional mitochondrial and nuclear multilocus sequence data. They identified three distinct ancestral lineages in the Andean region, with only one of these lineages was present in Mexico’s Toluca Valley [19]. Gómez-Alpizar et al. found no substantial indications of selection in the Toluca Valley but did detect evidence of a founder effect, suggesting Mexico as a region of secondary center of diversification of the pathogen [19]. The emergence of *P*. *andina*, with a shared common ancestry with *P*. *infestans* in the Andes, was cited as added support for a South American origin [19].

Fry revisited this controversy in 2008, portraying it as an unresolved inquiry due to conflicting evidence [16, 23]. In 2014, the Grünwald group revisited the center of origin subject, expanding their study to include additional isolates and three other *Phytophthora* species in the 1c clade, *P andina*, *P*. *ipomoeae* and *P mirabilis* [24]. That team used multilocus sequencing of a few loci and SSR datasets. The convergence of multilocus evidence for a Mexican origin, coupled with sexual reproduction in Mexico, a trait which is not geographically widespread in *P*. *infestans*, prompted them to assert Mexico as the center of origin [24]. However, their study did not include either an extensive global sampling of *P*. *infestans* or historic genomes. In 2016, the Ristaino lab with collaborators Mike Martin and Tom Gilbert, at the University of Copenhagen, conducted the largest whole genome sequencing project to date with historic and modern day lineages of *P infestans* [25]. Analysis of these more extensive genomic dataset that included both *P*. *infestans* and *P*. *andina* isolates documented an Andean origin of the species [25]. Lineages of Andean origin were found to be more closely related to historical *P*. *infestans* lineages from the famine era, implying an Andean origin with later subsequent migration and diversification occurring in Mexican lineages [25]. Significant admixture between the historic *P infestans* and *P andina* was also documented [25].

The unresolved dispute encircling the center of origin for *Phytophthora infestans* is underscored by studies such as those conducted by Knaus et al. (2020) and Martin et al. (2016) [25, 26]. For instance, while sexual recombination is regarded as evidence for a Mexican origin, *P*. *infestans* is mostly asexual and does not widely engage in sexual reproduction, despite the migration of the A2 mating type into Europe [1, 26]. In Europe, sexual populations have only been reported in the Netherlands and parts of Scandinavia even though both mating types are widely dispersed [1]. Recent research has demonstrated that *P*. *infestans* can undergo ploidy reduction in response to stress from anthropogenic sources such as fungicides or low nutrients, potentially facilitating sexual reproduction [27]. Both Mexican and South American populations of *P*. *infestans* manifest considerable diversity, rendering them both centers of diversity. The determination of the primary center of origin hinges on the time of divergence, underscoring the important role of population genomics and ancestry analysis with historic samples in addressing this question.

Progress in *Phytophthora* research has ushered in novel methodologies for probing the origins of *Phytophthora infestans*. Whole-genome sequencing has been performed by our lab and others on closely related 1c clade species, along with an array of *P*. *infestans* isolates (S1 Table in S1 File). The discovery of *Phytophthora urerae*, a newly described member of the 1c clade found in South America, has broadened the scope of species for phylogenomic analysis [11]. We have taken a pangenomic approach to study the genetic diversity of the *Phytophthora* 1c clade species by combining the genomes of multiple individuals. Our dataset includes the entire set of genes from all the species within the 1c clade. The incorporation of close kin of *P*. *infestans* from both Mexico and South America, and subpopulations of *P*. *infestans* from global modern and historic lineages in an analysis at the whole-genome level, holds potential for providing important data to identify the center of origin of the entire clade and *P*. *infestans* itself.

Our objective was to investigate *Phytophthora infestans’* origins through a pangenomic analysis of *P*. *infestans* and its 1c clade sister species. Our aim was to examine the genetic diversity among 1c clade species, trace ancestry and migration patterns between the species. Additionally, we examined ancestry and migration within various subpopulations of *P*. *infestans* beginning with the oldest historic famine lineage. To support the Mexican origin hypothesis, we would expect an ancestral history of genetic diversity within Mexican *Phytophthora* populations and migration out of Mexico of *P*. *infestans* over time. Conversely, if the Andean origin hypothesis held, we would anticipate ancestry there and migration of *P*. *infestans* originating from the Andean region. Unlike previous research that examined genetic diversity using limited multilocus nuclear loci and mitochondrial genome data, our study employed whole genome-level data from a global set of multiple isolates, including representatives of all the currently reported 1c clade *Phytophthora* species and the historic *P*. *infestans* lineage to explore evolutionary relationships. Our migration analysis utilized an expanded genomic dataset, distinct from prior studies, enhancing the depth of our investigation.

## Results

### Sample collection

We analyzed sequence data from isolates of all seven 1c clade *Phytophthora* species, totaling 69 samples, fourteen of which were sequenced in the present study, while others were sequenced in our or others previous work [25] (S1 Table in S1 File). These samples were subsequently categorized into 11 populations for analysis including the seven 1c clade *Phytophthora* species including *P*. *andina*, *P*. *betacei*, *P*. *infestans*, *P*. *ipomoeae*, *P*. *mirabilis*, *P*. *phaseoli* and *P*. *urerae*, and five subpopulations of *P*. *infestans* consisting of Historic, US-1, South America, Modern (US_8, US_22, US_23 and EU_13) and Mexican populations.

### Network and phylogenetic analyses for population differentiation

**Fig 1:**
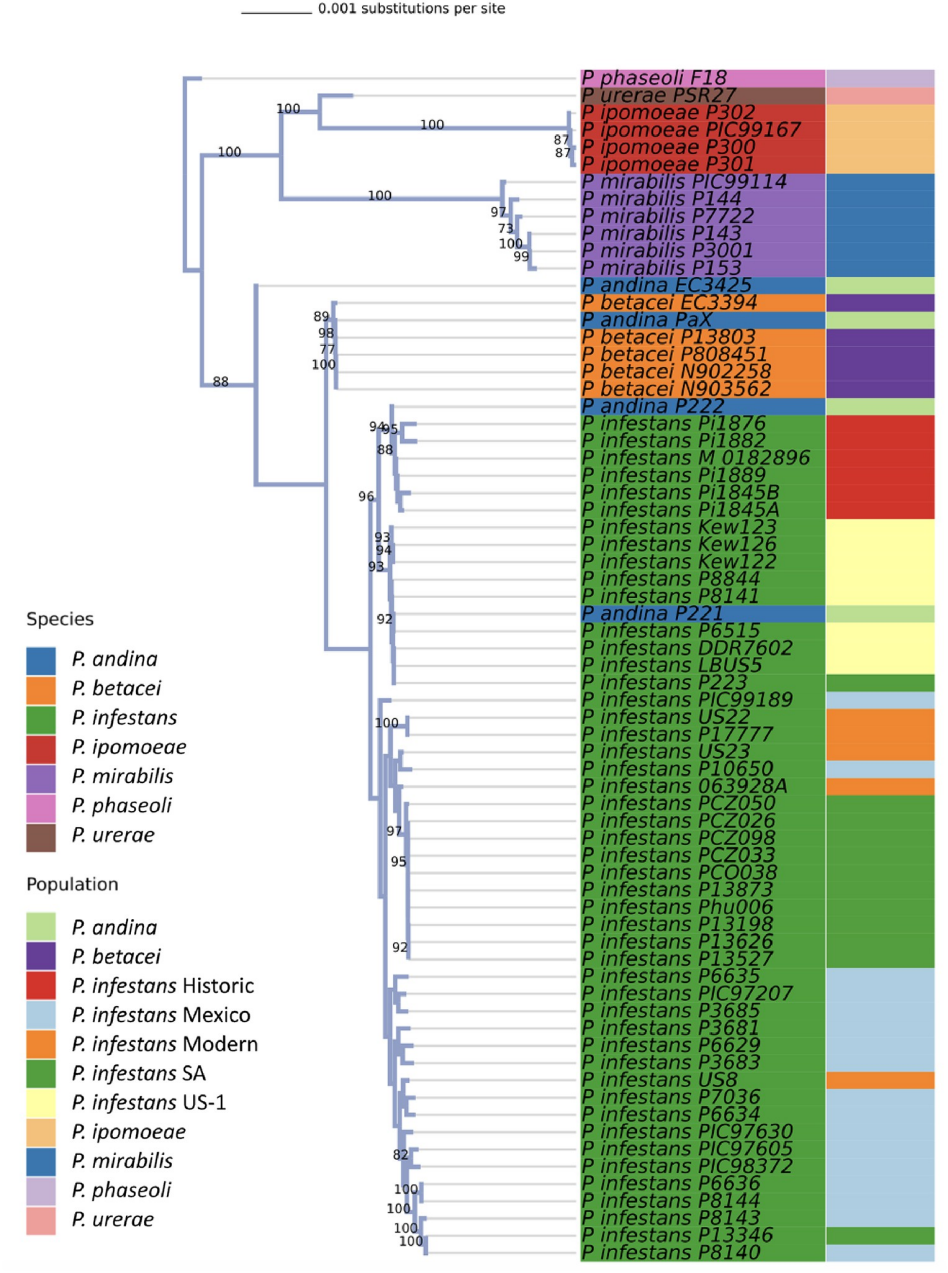
The phylogenetic tree of all *Phytophthora* 1c clade species included in this study, inferred with RAxML from genic regions across the genomes that were present in at least 90% of samples. Bootstrap values above 70% are shown. Leaves are colored according to species and the outer band is colored according to the source of population for five *P*. *infestans* subpopulations of isolates (Historic, US-1, South American (SA), Mexican and Modern).

Network and phylogenetic analyses were used to analyze the data for species within the 1c clade and *P*. *infestans* subpopulations, laying the foundation for subsequent migration analysis. The maximum likelihood tree supported *P*. *mirabilis* and *P*. *ipomoeae* as distinct monophyletic groups (Fig 1). *Phytophthora infestans* also formed a monophyletic group with the exception of two *P*. *andina* isolates (P221 and P222) that we found were misidentified in the Gallegly collection and are actually *P*. *infestans* (S1 Table in S1 File). Both were mitochondrial haplotype Ia and not Ic like the true *P*. *andina*. In this phylogeny, *P*. *betacei* formed a clade with the remaining true *P*. *andina* samples (PP3425 and Pax) (Fig 1). Historic *P*. *infestans* grouped together followed by US-1 *P*. *infestans*, SA, Mexican and modern *P*. *infestans* as expected, albeit with some outliers, particularly evident in *P*. *infestans* Modern and Mexican populations.

**Fig 2:**
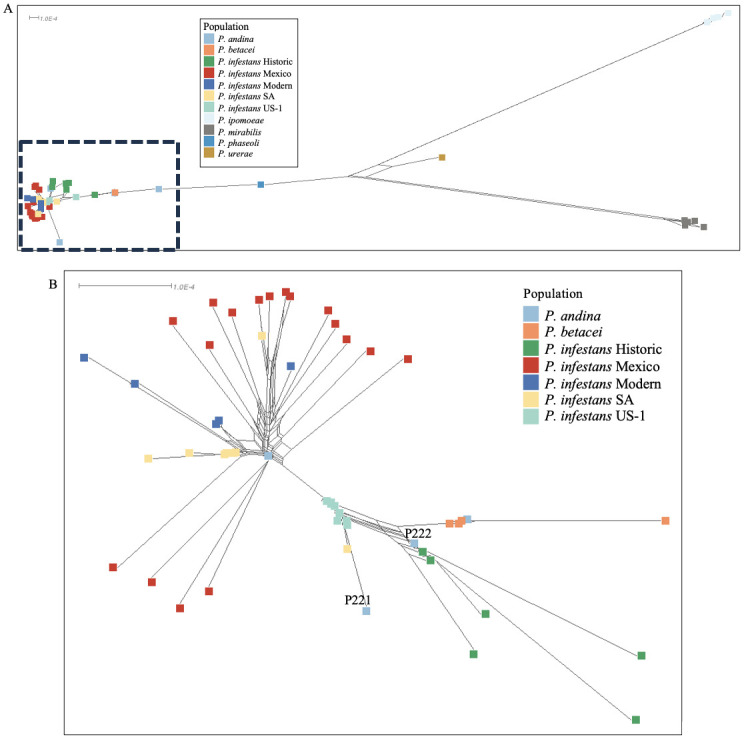
**A,** NeighborNet network created and visualized with SplitsTree. The network is divided into three clusters based on 3,485,475 SNPs. A, The first group includes *P*. *andina*, *P*. *betacei*, and *P*. *infestans* within a short distance from each other (lower lefthand corner) while *P*. *ipomoeae* (lightest blue, upper right) and *P*. *mirabilis* (gray, lower right) form two additional and distant clusters. *P*. *phaseoli* and *P*. *urerae*, which are each represented by only one sample each, are between these three disparate clusters and distinct. **B**, Closer view of NeighborNet network visualization of the distances between subpopulations of *P*. *infestans* and closely related species *P*. *andina* and *P*. *betacei* estimated and visualized with SplitsTree as shown in A.

NeighborNet graphs were also developed (Fig 2A and 2B). In a NeighborNet graph centered solely on the *P*. *andina*, *P*. *betacei*, and *P*. *infestans*, *P*. *infestans* again mostly clustered with the same exceptions as noted above for Modern and Mexican populations (Fig 2B). Importantly, *P*. *infestans* historic samples were the closest *P*. *infestans* population to *P*. *andina* and *P*. *betacei*. A SplitsTree network, encompassing all 69 samples within the dataset, unveiled three distinct clusters: 1) *P*. *ipomoeae*, 2) *P*. *mirabilis*, and 3) a complex of *P*. *andina–P*. *betacei–P*. *infestans* (Fig 2A). *P*. *phaseoli* and *P*. *urerae* occupied intermediate positions between these three clusters. Albeit, we only had one genome of these 2 species in this this analysis.

### Variant calling

Through amalgamating raw data from diverse sequencing experiments and harnessing publicly available genome sequencing data, in addition to conducting our own sequencing, we attained satisfactory sequencing depth for most samples: among the sixty-nine samples, sixty-two have a mean sequencing depth greater than 10X (S2 Table in S1 File, S1 Fig). Mapping percentages were comparatively lower for historic samples, and the four genomes sequenced using MinION sequencing demonstrated reduced mean base quality scores (S2 Table in S1 File).

Following the GATK best practices and implementing filtering for depth, quality, and minimum allele frequency using BCFTools, a grand total of 3,485,475 SNPs were identified throughout the dataset. The number of SNPs found in *P*. *infestans* isolates ranged from approximately 350,000 to 750,000 and was lower than the number of SNP’s found in the other 1c clade species (over 1 million). This is expected as the *P*. *infestans* T30-4 genome was used as the mapping reference for all isolates (S2 Fig). The ratio of sites harboring heterozygous SNPs (one allele matching the reference and one alternative allele) to homozygous SNPs (two alternative alleles) was higher within *P*. *andina*, *P*. *infestans*, and *P*. *betacei* compared to *P*. *ipomoeae* and *P*. *mirabilis* (S3A Fig). Transition-to-transversion (Ts/Tv) ratios across the dataset exhibited a higher degree of consistency, however, a few historic *P*. *infestans* samples displayed an elevated Ts/Tv value (S3B Fig).

### Nuclear diversity analysis

An assessment of nuclear diversity within the 1c clade species was conducted within each population. The average number of pairwise differences, nucleotide diversity, the count of segregating sites, and Watterson’s Theta demonstrated higher values for *P*. *andina* across coding regions compared to other 1c clade *Phytophthora* species (S4 Fig). Conversely, Tajima’s D was significantly larger than zero for *P*. *infestans* (p<0.0001), implying balancing selection or population contraction.

When exploring subspecies populations within *P*. *infestans*, it became evident that the average number of pairwise differences, nucleotide diversity, number of segregating sites, and Watterson’s Theta were higher for the Mexican and Modern populations than the rest of the populations (S5 Fig). Additionally, Tajima’s D exhibited elevated mean values for the Mexican, South American, and Historic populations in contrast to US-1 and Modern populations.

### Assessing population structure with ADMIXTURE

Population structure was analyzed using ADMIXTURE, with cross-validation plots indicating that four ancestral populations (K = 4) was the optimal choice (S6 Fig). Beyond K = 4, particularly at K = 7 and higher, we observed an increase in cross-validation error variance. At K = 4, isolates of *P*. *ipomoeae* and *P*. *mirabilis* formed distinct clusters corresponding to their respective populations (S7A Fig). Consistent with the maximum-likelihood phylogeny, the *P*. *andina*, *P*. *betacei*, and *P*. *infestans* group divided into two clusters, with all *P*. *betacei* isolates clustering within one of these. As K increased to 7, we noted further differentiation in population structure, especially among certain *P*. *infestans* isolates (S7B Fig).

### Migration analysis

The topology of the clade 1c phylogeny inferred from a concatenation of loci using RAxML (Fig 1) only differed slightly from a multilocus coalescence-based method using BEAST2 (S8 Fig). Because BEAST2 does not estimate the magnitude and direction of cross-species gene flow or introgression, which is a driving force in *Phytophthora* clade 1c, we used Ima3, a more complex model that estimates introgression, population sizes, and divergence times.

Both TreeMix and IMa3 (Isolation-with-Migration) were employed to infer migration patterns among the 1c clade species and *P*. *infestans* subpopulations [28, 29]. To ascertain the optimal number of migration edges (m) within the TreeMix model, iterative runs of TreeMix were conducted, encompassing a range of m values. OptM was then utilized to estimate the likelihood of m [30]. For the model featuring each 1c clade species as an individual population, m = 1 emerged as the optimal number of migration edges (S9 Fig). Within the species-level model, *P*. *ipomoeae*, *P*. *mirabilis*, and *P*. *urerae* formed one clade, while *P*. *andina*, *P*. *betacei*, and *P*. *infestans* comprised another (S10 Fig). A single migration event from the outgroup *P*. *phaseoli* to the common ancestor of *P*. *andina* and *P*. *infestans* was observed.

Another model, again encompassing all samples but further subdividing *P*. *infestans* into subpopulations, was also scrutinized. For this model, m = 2 stood out as the optimal number of migration edges (S11 Fig). In the model centered around *P*. *infestans* populations, the *P*. *andina–P*. *betacei–P*. *infestans* group underwent further partitioning into *P*. *infestans* populations. *P*. *infestans* populations US-1 and Historic *P*. *infestans* exhibited a closer affinity with *P*. *andina* and *P*. *betacei* (S12 Fig). Two migration events occurred: one from *P*. *infestans* Historic to the outgroup *P*. *phaseoli* and the other from *P*. *betacei* to *P*. *ipomoeae*.

For precise IMa3 outcomes, models were constructed to categorize samples into respective populations. Guided by the 1c clade phylogeny, network, and admixture results, distinct groupings were evident for *P*. *ipomoeae* and *P*. *mirabilis* (Figs 1 and 2). *P*. *urerae* and *P*. *phaseoli* were intermediaries, albeit each of these species are represented by a single sample in our study. The *P*. *andina–P*. *betacei–P*. *infestans* cluster exhibited both grouping and admixture across analyses. Informed by these insights, a three-population model was chosen for IMa3 migration analysis, with the three groups delineated as *P*. *ipomoeae*, *P*. *mirabilis*, and the species complex *P*. *andina–P*. *betacei–P*. *infestans*. Additionally, a detailed pairwise migration assessment was conducted among *P*. *mirabilis*, *P*. *andina*, *P*. *betacei*, and *P*. *infestans*.

For *P*. *infestans* populations, network, admixture, and phylogenetic analyses unveiled some isolates as outliers from their anticipated populations based on genotype and collection data, particularly noticeable in *P*. *infestans* Modern isolates (collected from 2006–2009) (Figs 1 and 2). All *P*. *infestans* Modern isolates, along with *P*. *infestans* SA and Mexico isolates that did not conform to their expected populations, were excluded from the *P*. *infestans* IMa3 analysis. These outliers are marked with asterisks in S1 Table in S1 File. Minus these outliers, the model comprised four populations: *P*. *infestans* Historic, *P*. *infestans* US-1, *P*. *infestans* South America, and *P*. *infestans* Mexico.

**Fig 3:**
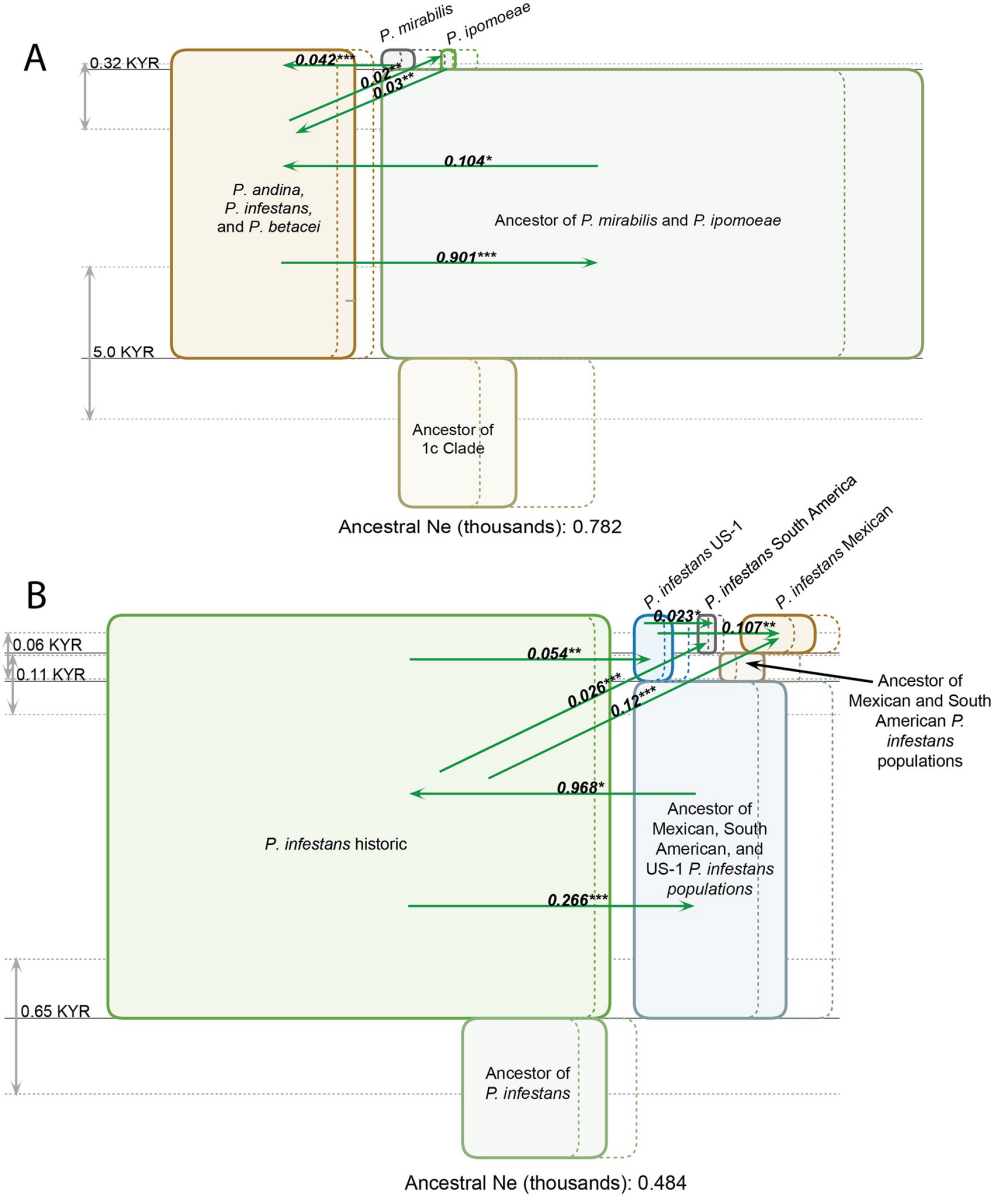
**(A)** Genealogy of *P*. *mirabilis*, *P*. *ipomoeae*, and the *P*. *andina-P*. *infestans-P*. *betacei* species complex inferred with IMa3 and visualized with the IMfig program. The phylogeny is depicted as a series of boxes organized hierarchically, with ancestor boxes positioned in between the corresponding descendants, and the width of boxes proportional to estimated N_e_ (effective population size). 95% confidence intervals for each N_e_ value are shown as dashed lines to the right of the left side of the corresponding population box. Splitting times are depicted as solid horizontal lines, with text values on the left. Confidence intervals for splitting times are shown as vertical gray arrows on the left, and parallel dashed lines. Migration arrows (if shown) indicate estimated 2Nm values from one population to another over the time interval when both populations exist. Arrows are shown only for estimated migration rates that are statistically significant at or above the 0.05 level (* p < 0.05, ** p< 0.01, *** p < 0.001. **(B)** Estimation of migration rates, time of divergence, and ancestral population size between five subpopulations of *P*. *infestans* inferred with IMA3 and visualized with IMFig. The *P*. *infestans* Modern population is not included because it was intermixed with other *P*. *infestans* subpopulations and difficult to differentiate in TreeMix.

The outcomes of the IMa3 analysis, based on the three-population model, indicated that the common ancestor of *P*. *mirabilis* and *P*. *ipomoeae* diverged from the *P*. *andina–P*. *betacei–P*. *infestans* cluster approximately 5 thousand years ago (Fig 3A). Notably, the *P*. *andina–P*. *betacei–P*. *infestans* cluster exhibited a larger population size compared to the other two species. This could be due in part to the large sample size of the *P*. *infestans* population used in this study, relative to the other species.

An IMa3 analysis targeting the *P*. *infestans* subpopulations showed more recent population divergence times (.65KYR) and smaller ancestral population sizes (N_e_ < 0.484 thousand), consistent with expectations at the subspecies level (Fig 3B). Migration emanated from the initial population divergence from Historic *P*. *infestans* to *P*. *infestans* US-1 populations, and from there to SA and Mexican *P*. *infestans*. Migration rates from Historic *P*. *infestans* to US-1, SA and Mexican populations were 0.54, 0.28 and 0.12, respectively. Migration rates from *P*. *infestans* US-1 to SA and Mexican populations were 0.23 and .107, respectively. All migration rates shown with arrows were significant at or above P < .05 (Fig 3).

**Fig 4:**
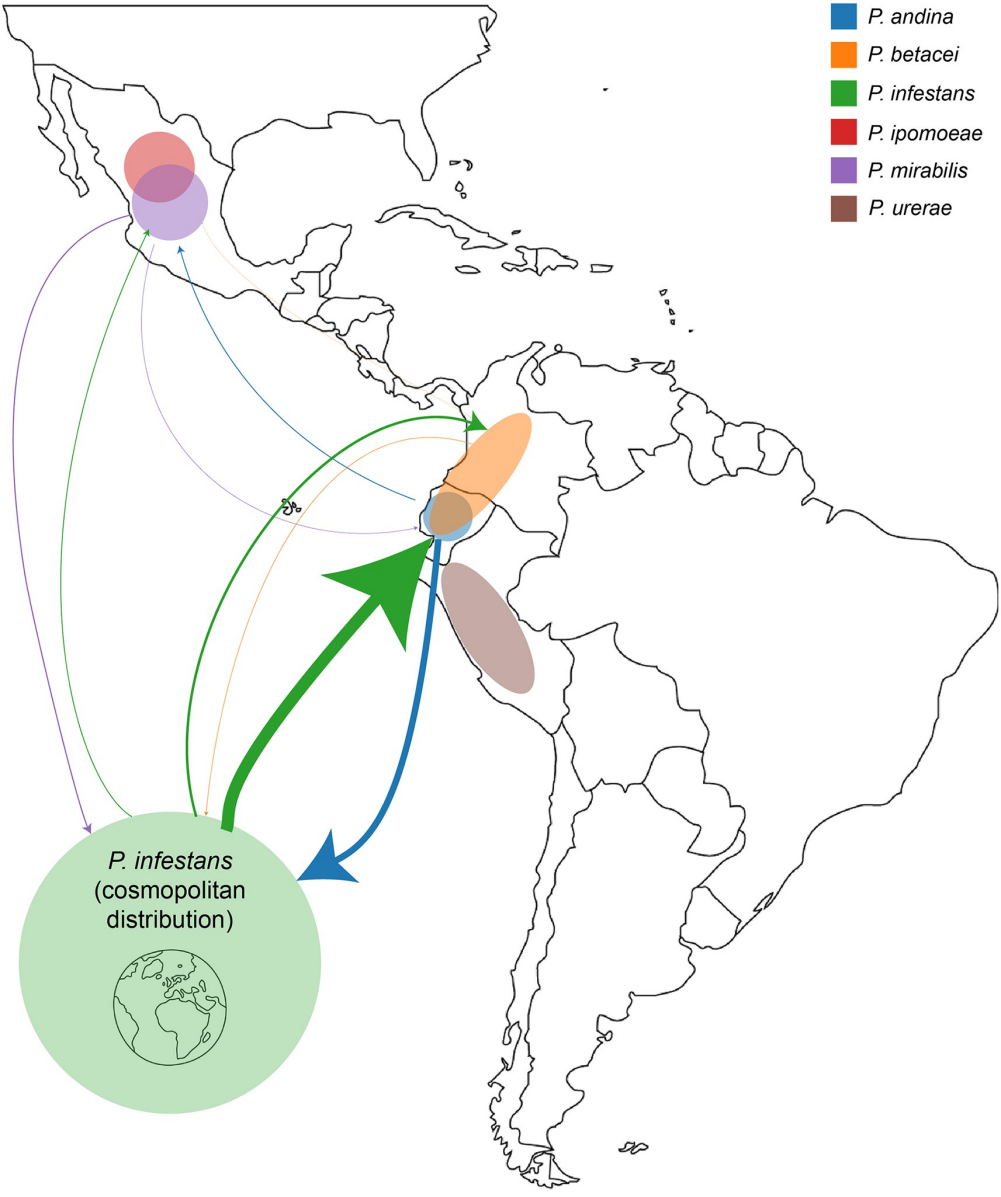
Map of 1c clade species (except for *P*. *phaseoli*) in Central and South America. Approximate distribution of countries in which each species can be found are shown with shaded circles (*P*. *infestans* is cosmopolitan). Migration rates estimated from pairwise IMa3 runs for each combination of species are shown for *P*. *infestans*, *P*. *andina*, *P*. *betacei*, and *P*. *mirabilis*. The thickness of each arrow is proportional to the rate of migration from one population to another by region. Map created using https://www.freeusandworldmaps.com, from Bruce Jones Design. 2004–2024.

Pairwise migration analysis with IMa3 between *P*. *andina*, *P*. *betacei*, *P*. *infestans*, and *P*. *mirabilis* along with their geographical distributions were summarized into a map, with weighted arrows representing migration rates (Fig 4, S3 Table in S1 File). The compilation of these IMa3 results shows that gene flow from the Mexican species *P*. *mirabilis* to the cosmopolitan *P*. *infestans* occurred at lower rates (0.009 versus 0.037 and 0.512, p<0.001 for all) compared to geneflow between *P*. *infestans* and South American populations of *P*. *andina* and *P*. *betacei*. It is important to note that gene flow into and out of the Andean region was higher than gene flow into and out of Mexico for *P*. *infestans*.

## Discussion

We compiled a pan-genome sequence data set from both experimental sources and public repositories for 69 *Phytophthora* isolates from the 1c clade (S1 Table in S1 File). Our objective was to examine the evolutionary relationships, origins, and migration patterns of *Phytophthora* species within the 1c clade. We used network and phylogenetic analyses, revealing that some *Phytophthora* 1c clade species are not as differentiated as expected. Notably, the three Andean species, *P*. *andina*, *P*. *betacei*, and *P*. *infestans* exhibited cohesive clustering in NeighborNet graphs, phylogenetic trees, and admixture graphs (Figs 1 and 2, S7 Fig). Meanwhile, *P*. *ipomoeae* and *P*. *mirabilis* consistently formed discrete more recent groups separate from the Andean species using diverse analytical methods, underscoring a clear divergence of these two species from the remaining Andean species in the clade. *P*. *infestans* appears to be very closely related to Andean localized species *P*. *betacei* and *P*. *andina*, but it is more distantly related to Mexican localized species *P*. *mirabilis* and *P*. *ipomoeae*.

Our initial focus was on establishing the evolutionary relatedness among various 1c clade populations as a precursor to investigating migration. Starting with the currently described seven 1c clade species, we segregated the 69 samples into corresponding groups. A NeighborNet graph, encompassing all 69 samples examined in this study, distinctly displayed the 1c clade forming three conspicuous clusters (Fig 1). Notably, isolates affiliated with either *P*. *mirabilis* or *P*. *ipomoeae* occupied distinct clusters, lending substantial support to considering these species as distinct. Across multiple analyses, *P*. *mirabilis* and *P*. *ipomoeae* formed distinct individual monophyletic subclades within the 1c clade.

However, the scenario differed for *P*. *andina*, *P*. *betacei*, and *P*. *infestans* isolates. The NeighborNet analysis failed to show monophyletic clades within each of these species. Instead, a complex web of reticulate evolution was revealed (Fig 2). This complexity received further confirmation from the admixture analysis, which could not reliably distinguish these three species, however some distinction between *P*. *infestans* and *P*. *betacei* was evident for K = 4 (S7 Fig). Although a phylogenetic tree constructed using maximum likelihood managed to separate *P*. *betacei* and *P*. *infestans* from each other, it still failed to separate both species from *P*. *andina* (Fig 1). A Bayesian analysis of these molecular sequences produced comparable results, showing some *P*. *andina* and *P*. *betacei* isolates forming an outgroup from *P*. *infestans* (S13 Fig). Since these three *Phytophthora* species infect some of the same hosts, both speciation and interspecies hybridizations are occurring on some of these hosts, so results are not surprising.

Two isolates of *P*. *andina*, P221 and P222, grouped amidst *P*. *infestans* isolates in multiple analyses (Figs 1 and 2). Based on our phylogenetic data as well as mitochondrial haplotyping of these isolates using methods of Oliva et al. (both isolates were mtDNA haplotype Ia, not haplotype Ic like true *P*. *andina*), we recommend moving these two isolates from *P*. *andina* to *P*. *infestans* [18]. They were misnamed in the M. Gallegly collection. Both *P*. *andina* and *P*. *betacei* exhibited elevated levels of heterozygosity, alongside an exceptionally high number of SNPs relative to the *P*. *infestans* reference genome (S2 and S3 Figs) [14, 31]. Intriguingly, in network analysis, *P*. *betacei* exhibited a close affinity to the Historic *P*. *infestans* and *P*. *infestans* US-1 populations, two of the oldest *P*. *infestans* populations in our dataset (Fig 2).

Our work and a closer examination of networks and phylogenies involving *P*. *infestans* population challenges prior assumptions about the subpopulations [17, 19, 24, 25, 32]. Despite the emphasis on separating South American and Mexican *P*. *infestans* populations, our data reveal that migration and gene flow is occurring between these two closely related subpopulations of *P*. *infestans* (Figs 3 and 4). Migration and gene flow from Historic to South American *P*. *infestans* and subsequent gene flow to Mexican *P infestans* populations was documented (Fig 3B).

Migration between *P*. *andina*, *P*. *betacei*, and *P*. *infestans* species was clear, with elevated migration rates when compared to other 1c clade species (Fig 3A, S3 Table in S1 File). The remarkably recent divergence times imply a recently evolving species complex. Our findings suggest intense migration among these 1c clade species endemic to the Andean region (*P*. *andina* and *P*. *betacei*) and *P*. *infestans*. These observations, combined with the recent divergence times (0.32–5 KYR), suggest both recent and ongoing speciation in the Andes (Fig 3A).

Looking at pairwise migration rates between *P*. *andina*, *P*. *betacei*, *P*. *infestans*, and *P*. *mirabilis*, the largest migration rates are within the *P*. *andina–P*. *betacei–P*. *infestans* group in South America (Fig 4). Migration rates to and from the Mexican species *P*. *mirabilis* were significantly lower. Migration between populations of *P*. *infestans* was from *P*. *infestans* Historic populations to US-1, SA and then Mexican lineages (Fig 3B). Given that *P*. *infestans* Historic population represents the oldest population sampled in this study (1845–1889), our data document gene flow between historic and recent populations in the last century [3, 4].

Numerous prior studies have tackled the enigmatic origin of *P*. *infestans* and the broader 1c clade, yielding conflicting conclusions [3, 17, 19, 24, 33]. Many of these investigations primarily leaned on limited multilocus mitochondrial and nuclear datasets, often with fewer samples from other 1c clade species and even fewer South American samples. Our study capitalized on a more comprehensive 1c clade dataset, coupled with an expansive nuclear genomic analysis, to illuminate the origin of *P*. *infestans* and its intricate connections to other 1c clade species. The central debate has revolved around whether the globally pervasive *P*. *infestans*, responsible for devastating outbreaks including the Irish Potato Famine, originated from Mexico or the Andean region of South America [16, 19, 21, 24, 25, 32, 34, 35].

By deploying an array of population genetics methodologies—ranging from admixture analysis and phylogenetics to nuclear diversity assessment to migration analysis—across the full spectrum of 1c clade genomes, a consistent narrative emerges. The divergence of the Mexican species *P*. *mirabilis* and *P*. *ipomoeae* from *P*. *infestans* is clear (Figs 1 and 2, S7 Fig). However, the separation of *P*. *infestans* from fellow 1c clade members *P*. *andina* and *P*. *betacei*, both confined to the Andean region of South America, is less distinct (Fig 2). This suggests that speciation of *P*. *infestans* and its close sister lineages, *P*. *andina* and *P*. *betacei* took place, and continues to take place, within the Andean region of South America.

We previously reported that historic *P*. *infestans* populations derived from mycological herbarium collections share admixture with *P*. *andina* found in the Andes [19, 25]. We clearly document that once again in our work here. We also have documented that more recent populations of *P*. *infestans* in SA, Mexico and elsewhere migrated from Historic *P*. *infestans* (Fig 3B). In addition, migration rates out of and into the Andean region are larger than out of and into Mexico (Fig 4). Our data strongly support the Andean origin of *P*. *infestans*, the 1c clade and the historic FAM-1 strain that caused the Irish famine outbreaks. A recent study published using SSR genotyping to study global populations of *P*. *infestans* and has reached the similar conclusions, that *Phytophthora infestans* originated in the Andean region of South America [33].

Our findings reveal that contemporary Mexican *P*. *infestans* and South American *P*. *infestans* lineages trace their ancestry back to Historic *P*. *infestans* found in 19^th^ century herbarium specimens (Fig 3B). Moreover, the Mexico *P*. *infestans* and South American *P*. *infestans* populations exhibit striking genetic similarity across multiple metrics (Figs 1 and 2). Notably, the demarcation between Mexican and South American lineages, though emphasized by previous research, appears less pronounced in light of admixture patterns among more recent populations of *P*. *infestans* and the distribution of Modern *P*. *infestans* lineages across these groups [3, 5, 17, 19, 24, 25]. The intermixing of *P*. *infestans* in these two regions could even have occurred before *P*. *infestans* expanded to other parts of the world from an Andean source as both Indigenous and colonizing peoples moved potatoes from South to Central America and Mexico.

Furthermore, differentiating between *P*. *infestans*, *P*. *andina*, and *P*. *betacei* is not as straightforward as discerning other 1c clade species. Given distinctive attributes, such as *P*. *andina*’s hybrid nature and *P*. *betacei’*s host specificity, categorizing them as separate species remains useful for both taxonomic and regulatory reasons. However, our analysis and others suggests that *P*. *andina* is not monophyletic and has undergone admixture with *P*. *infestans* [17, 25]. Nothospecies or recent hybrids, are generally not monophyletic. Similarly, *P*. *betacei* exhibits recent admixture with both *P*. *infestans* lineages and *P*. *andina*. In this context, viewing these three species as an intricate species complex—characterized by nondistinct boundaries and ongoing admixture—offers a more accurate representation.

The rationale behind previously categorizing *P*. *andina*, *P*. *betacei*, and *P*. *infestans* as independent species rather than a species complex is worth contemplating. *P*. *andina* was initially identified as a hybrid of *P*. *infestans* and an unidentified species, and the initial description of *P*. *andina* encompassed EC-3 lineage samples now classified as *P*. *betacei* [14, 17, 25, 31]. Furthermore, *P*. *betacei* was previously presumed to have emerged from *P*. *infestans* through processes such as whole genome duplication and/or transposable element invasion [31]. These species were recognized to share high similarity and differentiation was primarily predicated on mitochondrial haplotypes and host range.

However, upon scrutinizing nuclear data in addition to mitochondrial data, these distinctions appear less clear. Favoring nuclear data over mitochondrial data proves advantageous for the phylogenetic and population analysis of *Phytophthora* due to mitochondria’s uniparental inheritance pattern, which can yield different outcomes in phylogenies. We chose to examine both nuclear and mitochondrial phylogenies here. A phylogeny inferred from mitochondrial loci also failed to differentiate these three species here (S13 Fig). Others have reported progeny resulting from crosses between both *P*. *infestans* and *P*. *betacei*, as well as *P*. *infestans* and *P*. *andina*, further underlining the possibility of gene flow between these closely related species [14, 17, 25, 31].

In future work, it remains paramount to sustain the sampling efforts focused on the *P*. *andina–P*. *betacei–P*. *infestans* species complex in South America. Exploring potential recombination events and genetic exchange within and between these groups holds significance, given their potential influence on shifts in virulence or host range. Additionally, it is imperative to delve deeper into the genetic disparities present among these taxa. Particularly, the pronounced heterozygosity exhibited by *P*. *andina* and the substantial number of SNPs in *P*. *betacei* warrant more thorough investigation.

One plausible explanation for these disparities is the occurrence of repeated hybridizations among lineages within this intricate complex. Such occurrences, as suggested by the network analysis, could entail divergent lineages coming into contact (Fig 2). Alternatively, the genetic interchange within this species complex might not be as straightforward as initially presumed. Avenues such as lateral gene transfer or alterations in ploidy could account for these observed genetic variations. While *P*. *andina* maintains diploidy, the modern aggressive *P*. *infestans* lineages are triploid, and sexually recombining lineages are diploid [25]. *P*. *betacei* has been recognized for possessing a significantly larger genome than *P*. *infestans* [14, 31]. Interestingly, recent studies have highlighted the prevalence of aneuploidy in *P*. *infestans* [36]. A plausible hypothesis could be that the variations between these isolates are tied to their respective chromosome counts. While we endeavored to evaluate the ploidy of the isolates featured in our study, distinguishing between diploidy and triploidy with a high degree of confidence remained challenging for most isolates based on SNP data alone. This predicament could also potentially be attributed to aneuploidy.

Our data have documented an Andean origin of the entire 1c clade of *Phytophthora* and the historic lineage that caused the famine. We do know from haplotyping *P*. *infestans* infected herbarium samples that the famine lineage was present in Colombia in 1913 [4]. Historic *P*. *infestans*, samples collected from 1845–1889, were the first to diverge from all other *P*. *infestans* populations, with modern South American and Mexican populations both showing shared ancestry derived from historic *P*. *infestans*. Based on the time of divergence of *P*. *infestans* from its closest relatives, *P*. *andina* and *P*. *betacei*, in the Andean region, we consider the Andes to be the center of origin of *P*. *infestans*, with modern globalization contributing to admixture between *P*. *infestans* populations today from both the Mexican and Andean regions.

## Materials and methods

### DNA preparation and sequencing

A combination of sequence data was mined from publicly available sources or generated by our team in this or previous work (S1 Table in S1 File). Thirteen isolates were sequenced at the Norwegian University of Science and Technology (NUST), Trondheim, Norway (S1 Table in S1 File). Sequencing of the *P*. *urerae* DNA was done at NC State in a paired-end format using an Illumina NovaSeq platform. Seven species of *Phytophthora* from the 1c clade were grown in pea broth. DNA was first extracted from these isolates using the CTAB method from mycelial tissue [37]. DNA extracts were sheared to a mean fragment length of 550 bp using the Covaris ME220 focused ultrasonicator. Then the sheared DNA samples were built into double-stranded genomic libraries using the BEST library preparation method [35]. Indexing PCR was performed using custom dual indexing primers [36] in 100-μL reactions with 7.5 μL unamplified library, 2.6 μL of 10-μM forward primer, 2.6 μL of 10-μM reverse primer, 1 μL Herculase II Fusion DNA polymerase, 20 μL 5X Herculase II Reaction Buffer, and 65.3 μL molecular biology H2O. The thermal cycling conditions were 3 min at 95°C, 8–16 cycles of 20 sec at 95°C, 20 sec at 60°C, 40 sec at 72°C, and final extension for 5 min at 72°C. The amplified libraries were purified with a 1:1 ratio of SPRI beads [37] and eluted in EB buffer. The purified libraries were quantified on the Agilent 4200 TapeStation automated electrophoresis system, pooled equimolarly, and then sequenced on the Illumina NovaSeq 6000 platform, generating 100-bp paired-end reads.

Additionally, we sequenced four isolates from different 1c clade species (*P*. *infestans* RS2009P1; *P*. *mirabilis* P144; *P*. *ipomoeae* PIC99167; *P*. *phaseoli* F_18), using long-read sequencing and the Oxford Nanopore MinION platform with a R9.4.1 flow cell and the SQK-LSK110 Ligation Sequencing Kit. DNA extractions for the MinION sequences were performed with a QIAGEN DNeasy Plant Mini Kit following the manufacturer’s instructions (Qiagen, Valencia, CA). Sequencing was done with the Ligation Sequencing Kit (SQK-LSK110), Agencourt AMPure XP beads (Beckman Coulter, A63881), and the NEBNext Companion Module for Oxford Nanopore Technologies Ligation Sequencing (E7180S) following the manufacturer’s instructions.

Sequencing strategy for the publicly available samples from previous work varied between paired-end and single-end Illumina libraries depending on the sample. Additionally, one sample, *P*. *betacei* P8084, was sequenced with the PacBio long read format [31]. Data from both short read and long read projects were retrieved for these samples. References for publicly collected data and associated metadata are shown in S1 Table in S1 File.

### Read trimming and alignment

All raw reads from sequencing experiments on Illumina and PacBio platforms, whether publicly available or collected as part of this study, were trimmed. Trimming was performed using TrimGalore v0.6.10, a wrapper around Cutadapt v4.0 and FastQC v0.12.1 [38–40]. Adapters were removed, along with reads that were less than 20 base pairs in length. A quality cutoff of 20 Phred score was applied. For specific samples that were sequenced on NovaSeq or NextSeq platforms (all samples sequenced as part of this study), the “-nextseq" quality flag within Cutadapt was utilized to prevent the potential G overcalling associated with these platforms. FastQC results for the trimmed reads were consolidated using MultiQC v1.12 and then reviewed manually [41]. Following manual review, a second round of trimming was conducted on certain samples to eliminate spurious poly-A reads. In the case of long reads generated by MinION sequencing, reads with a quality score below 8 were filtered out using MinKNOW v22.05.5.

Subsequently, the trimmed reads were aligned to the reference genome assembly T30-4 of *Phytophthora infestans*, as well as the *P*. *infestans* mitochondrial genome reference haplotype Ib [42, 43]. Alignments for nuclear and mitochondrial sequences of each sample were kept separate to facilitate downstream analysis specifically focused on nuclear genomes. For most short read Illumina accessions and all newly sequenced Illumina runs, the BWA-MEM algorithm from the Burrow-Wheelers Aligner v 0.7.17 was employed to align the reads to their respective genomes [44]. For a few samples originating from older Illumina sequencing projects with shorter average read lengths, the BWA-ALN algorithm followed by BWA-SAMSE were used for alignment. This approach was taken to adhere to best practices that consider the appropriate algorithm based on read lengths. In the case of all long reads generated using PacBio or Oxford Nanopore technologies, Minimap2 v2.24 was employed for aligning the reads to the reference genome.

### Variant calling

To effectively group reads for subsequent variant calling, read groups were introduced into each alignment using Picard v2.3.1’s AddReadGroups tool [45]. These read groups provided specifications for the sample, experiment, and library associated with each alignment. Subsequently, all alignments underwent sorting through SAMTools v1.16.1 [46]. The Picard MarkDuplicates tool was employed to identify any PCR-generated duplicates stemming from the library preparation process [45]. Any duplicates attributed to amplification rather than biological duplication were disregarded during the variant calling phase.

Haplotypes were invoked using GATK (version 4.2.6.1) HaplotypeCaller in ERC mode, yielding GVCF (Genomic Variant Call Format) files that encompassed genotype likelihoods [47]. All GVCF haplotype call files were merged using GATK GenomicsDBImport to construct an indexed database. Following this, joint genotyping for each cohort was executed using GATK GenotypeGVCFs, culminating in a unified VCF (Variant Call Format) file that encompassed genotypic information. The VCF file encompassing all samples was subsequently divided into two categories: INDELs and SNPs. Both INDELs and SNPs underwent rigorous hard filtering to eliminate any low-quality variants.

### Alternative genome sequences

After identifying and filtering the variants across all samples, the identified SNPs were applied to the reference genome to generate alternative genome sequences for each individual sample. Initially, mask files were created for each sample to obscure regions with low coverage from being present in the alternative genomes. The BEDTools v2.27.1 suite was utilized to determine the genomic positions covered by a minimum depth of 10 reads [48]. Any positions with coverage less than 10 were recorded in a BED file. This BED file served as an input mask in conjunction with BCFTools v1.10.2 consensus, allowing low coverage regions to be masked out for each sample [46]. BCFTools consensus incorporated the processed SNPs, the reference genome, and the sample’s mask to produce an alternative FASTA file containing both applied variants and masked low coverage regions. Heterozygous sites were represented using IUPAC ambiguity codes. The same process was repeated for all mitochondrial genomes.

### Sequencing depth and variant calling analysis

To evaluate the sequencing depth of each sample, the SAMTools suite was employed. SAMTools stats, flagstat, and coverage functions were executed on each alignment file, revealing the count of reads aligned to both the reference nuclear genome and mitochondrial genome from the sequencing libraries. Utilizing these tools, various coverage statistics were aggregated for each of the alternative genome sequences [46].

BCFtools stats was utilized to generate summary statistics for the filtered VCF file containing only SNPs across all samples [46]. These statistics were compiled and then visualized using Microsoft Excel.

### Nuclear diversity analysis

The diversity levels within each population were assessed by calculating metrics including the average number of pairwise differences, nucleotide diversity, number of segregating sites, Tajima’s D, and Watterson’s Theta for each nuclear locus. These computations were conducted using a custom Python script that utilized the DendroPy v4.0 package [49]. Results of variant annotation with snpEff v8.32 were also used to count the number of missense and synonymous mutations per locus [50]. The outcomes of this analysis were consolidated into box plots through another custom Python script. To enhance visualization, a constant value was added to all measurements (to prevent zero values), and subsequent log transformation was applied. This comprehensive analysis was conducted across five species within the 1c clade: *P*. *andina*, *P*. *betacei*, *P*. *infestans*, *P*. *ipomoeae*, and *P*. *mirabilis*. Furthermore, the nucleotide diversity analysis was extended to encompass five *P*. *infestans* populations: Historic, Modern, US-1, South America, and Mexico.

### Network splits tree graph

The BEDTools getfasta function was applied, utilizing a BED file outlining the gene locations in the reference genome, to extract gene coding regions from each alternative genome sequence [48]. All alternative genome sequences were aggregated into a single FASTA file. Each entry in this file consisted of concatenated sequences of all genes for each sample, incorporating both masking of low coverage regions and applied SNPs. This process yielded an alignment encompassing coding regions for all samples. Subsequently, this FASTA-formatted file was transformed into a NEXUS-formatted file using the DeCIFR Toolkit [https://tools.cifr.ncsu.edu/sequence_converter]. The resulting alignment then underwent processing via TrimAl v.12, which entailed trimming regions where over 10% of samples exhibited missing data [49]. The outcome was a robust alignment of coding regions for each sample, balancing the preservation of data for analysis. The processed NEXUS file was introduced into the DeCIFR Toolkit’s SplitsTree implementation [https://tools.cifr.ncsu.edu/splitstree], where the NeighborNet algorithm was employed to gauge distances between lineages [50–52]. The resulting graph was then visualized using the desktop SplitsTree v4.19.1 application [51].

SplitsTree was also utilized in a similar fashion to deduce NeighborNet graphs for smaller subgroups within the sample set [50–52]. A reduced 1c clade sample set, encompassing data exclusively from *P*. *betacei*, *P*. *andina*, and *P*. *infestans* samples, underwent evaluation. Furthermore, an even more limited sample set exclusively containing subpopulations of *P*. *infestans* (Historic, Modern, US-1, South America, and Mexico) were created.

### Bayesian analysis of molecular sequences

The alternative FASTA sequences generated for all *P*. *infestans*, *P*. *andina*, and *P*. *betacei* isolates were also used as input for a Bayesian phylogenetic analysis. This sequence data, along with the date of collection and country of origin, were input into BEAUti v2.7.5 to generate input files for BEAST2 v2.7.5 with a chain length of 10 million [53]. Log normal priors were utilized for the relativeGeoRates and traitClockRate. A consensus tree from this multilocus, coalescence-based method with BEAST2 was developed with TreeAnnotator and visualized with FigTree v1.4.4 [53].

### Population admixture

The VCF file containing all high-quality, filtered SNPs across all samples was converted to binary PED format using VCFTools v0.1.16 [54]. This binary PED file, containing information about all variants filtered to remove linked SNPs, was then input into ADMIXTURE version 1.3.0, with the cross-validation flag activated to enable cross-validation analysis [55]. This process was iterated for values of K ranging from 1 to 15. The cross-validation results were graphed to visualize local minima, leading to the determination that K = 4 was the optimal value for the number of ancestral populations. The outcomes of the ADMIXTURE run were visualized using a customized Python script. Visual representations were generated for K = 4 (local minimum) and K = 7 (number of species).

### Maximum likelihood phylogenetic inference

The NEXUS alignment file generated for SplitsTree was also utilized to infer a phylogeny encompassing all samples. Additionally, an analogous NEXUS file was generated from alternative mitochondrial genome sequences to compare mitochondrial genomes. RAxML version 8 [56] implemented within the DeCIFR Toolkit [https://tools.cifr.ncsu.edu/denovo] and working seamlessly with CIPRES via the REST API service as employed to reconstruct the maximum likelihood phylogeny for the 1c clade based on the processed NEXUS alignments [57]. Node statistical support values (>70%) were based on 1000 bootstrap replicates under the GTRGAMMA model [58]. The resultant phylogenetic trees were visualized using T-BAS within the DeCIFR Toolkit [58, 59].

### Historical population analysis with admixture

The multi-sample, whole-genome VCF file containing filtered SNPs for all samples underwent processing with VCFTools and Plink v1.90b6.21 to eliminate sites with more than one sample missing or showing signs of linkage disequilibrium [54, 60]. A manual cluster file was created to define the species to which each sample belonged. VCFTools, Plink, and TreeMix were collectively used to convert the processed VCF file and the cluster file into TreeMix-formatted input [28, 54, 60]. For determining the appropriate number of migration edges to employ in TreeMix, results for three replicates for each potential number of migration edges, ranging from 0 to 7, were evaluated using the OptM web application [30]. TreeMix version 1.13 was used to estimate the tree, migration patterns, and residuals, employing 500 SNPs per block, bootstrapping, and using *P*. *phaseoli* as an outgroup [28, 30]. The small sample size correction was disabled.

In addition to the analysis conducted on the 1c clade species, a more detailed investigation was conducted with *P*. *infestans* divided into five subpopulations. OptM was again applied to estimate the number of migration edges [30]. In this case, three replicates were used for each potential migration edge count, ranging from 0 to 11. Otherwise, the same TreeMix parameters as described above were utilized [28].

### Isolation with migration analysis

The Py-Popgen-Pipeline was employed to process the filtered SNPs across all samples and prepare them for migration analysis [61]. Initially, a manually created model file designated which samples corresponded to specific populations. Subsequently, this model file and the SNP variants for all samples were filtered to include only biallelic sites where less than 10% of data was missing. Following this, a BED file delineating the location of all nuclear loci was utilized to identify informative sites, leading to the selection of a random subset of 200 loci. The processed VCF file was then partitioned into 200 separate VCF files, each pertaining to an individual locus. These locus-specific VCF files were phased and subsequently subjected to the four-gamete test to ensure their validity. For each locus, a random interval devoid of recombination was determined. These processed regions, along with the reference genome, were combined to create the input file for IMa3 v 1.12, assuming an inheritance scalar of 1.

The process elucidated in the paragraph above for generating IMa3 input files was repeated for various sample sets obtained from published genomes shown in S1 Table in S1 File [62–65]. Initially, based on the SplitsTree NeighborNet graph inferred for all samples, the dataset was divided into three groups: *P*. *ipomoeae*, *P*. *mirabilis*, and P. *andina-P*. *infestans-P*. *betacei*, where the latter group encompassed samples from all three species: P. *infestans*, *P*. *andina*, and *P*. *betacei*. Another sample set involved a model of four distinct *P*. *infestans* populations: *P*. *infestans* Mexico, *P*. *infestans* South America, *P*. *infestans* Historic, and *P*. *infestans* US-1. The *P*. *infestans* Modern population was excluded due to findings from SplitsTree NeighborNet graphs and other analyses indicating that the *P*. *infestans* Modern samples did not cluster together. Furthermore, certain *P*. *infestans* samples that deviated from their expected population in the SplitsTree NeighborNet analysis, and the maximum likelihood phylogeny were excluded because they could not be unambiguously assigned to a population. Additionally, two-population models were configured for every combination of *P*. *andina*, *P*. *betacei*, *P*. *infestans*, and *P*. *mirabilis* to elucidate migration patterns between these populations.

For models involving at least three populations (the *P*. *ipomoeae*/*P*.*mirabilis*/*P*. *andina-P*. *betacei-P*. *infestans* model and the *P*. *infestans* populations subset model), the initial IMa3 run was utilized to infer the best tree topology. IMa3 was executed through the implementation available in the DeCIFR Toolkit [https://tools.cifr.ncsu.edu/ima3] [29]. All runs were performed through the REST API service at CIPRES using program calls from IMa3 [57]. For topology inference, a burn-in of 1,000,000 steps was followed by a recording period of 10,000 phylogenies, with recording occurring every 100 steps. Maximum population size, maximum time of population splitting, and migration prior value were set to 2, 1, and 1, respectively, as prior estimates. A geometric heating scheme of -ha 0.998 and -hb 0.4 was applied across 256 chains, following recommendations from the IMa3 manual. The -j0 and -j3 flags were employed to estimate topology with hyperpriors as advised. Output was scrutinized to ensure high swap acceptance rates (> 0.95) between successive heated chains, high effective sample sizes (ESS > 10,000) and no obvious trends in trend plots, which all point to satisfactory mixing. The resulting topologies were consistent with the topologies previously inferred using maximum likelihood for the same populations.

After determining topologies, or for models involving only two populations and thus lacking topology, the same parameters were utilized to infer migration ancestral population sizes and splitting times. Demographic parameters were estimated assuming a mutation rate of 1e-06 substitutions per base per year and a sexual generation time of 1 year. Results were replicated three independent times for each model to assess convergence of parameter estimates. IMFig within the DeCIFR Toolkit was used to visualize the results [https://tools.cifr.ncsu.edu/ima3].

## Supporting information

S1 Fig Mean depth of coverage per sample across the T30-4 reference genome based on the aggregate number of mapped base pairs for each sample. (TIF)

S2 Fig The number of filtered SNPs in each sample with over 10X mean coverage depth. (TIF)

S3 Fig Ratio of heterozygous sites to homozygous sites and transition to transversion ratio for each *Phytophthora* species sample. **(A)** Ratio of heterozygous sites (1 reference allele, 1 alternative allele) compared to homozygous alternate sites (2 alternate alleles). All samples with an average coverage depth at variant sites above 10X are included. **(B)** Transition to transversion ratio for each sample. All samples with an average coverage depth at variant sites above 10X are included.
(TIF)

S4 Fig Summary of average number of pairwise differences, nucleotide diversity, number of segregating sites, Tajima’s D, Waterson’s Theta, and ratio of missense /synonymous mutations for each of the species’ sequences (>1 isolate). Data is log-transformed to facilitate visualization.
(TIF)

S5 Fig Summary of pairwise differences, nucleotide diversity, number of segregating sites, Tajima’s D, Waterson’s Theta, and ratio of missense /synonymous mutations for each population of *P*. *infestans*. Data is log-transformed to facilitate visualization.
(TIF)

S6 Fig Cross-validation plot of different values of K using ADMIXTURE. The local minimum around K = 4 shows this to be a reasonable modeling choice. When K> = 7 the variation in the cross-validation increased significantly. K = 7 (the number of recognized species in the dataset) and K = 14 (another local minimum) was also plotted to investigate population groupings within the dataset.
(TIF)

S7 Fig ADMIXTURE plots of 1c clade species and subpopulations of *Phytophthora infestans*. ADMIXTURE plots shown for (**A**) K = 4 and (**B**) K = 7. Bars indicate the proportion of genome ancestry from each ancestral population (K). Brackets are shown to represent a simplified breakdown of where each defined population can be found in relation to the ADMIXTURE generated populations.
(TIF)

S8 Fig Summary tree of Bayesian phylogenetic reconstruction of *P*. *infestans*, *P*. *andina*, and *P*. *betacei* using BEAST2 and visualized with FigTree. Branches are color coded according to region of origin and branch widths are proportional to posterior support for the branch. The tree is rooted with the *P*. *betacei/P*. *andina* clade as the outgroup.
(TIF)

S9 Fig TreeMix model results for numbers and rate of change of migration edges for 1c clade species. **(A)** Estimation for the optimal number of migration edges when considering each *Phytophthora* 1c clade species as a population with the TreeMix program, developed using OptM. **(B)** Based on the second-order rate of change in likelihood (Δm), 1 is the optimal number of migration edges for a TreeMix model using a dataset comprised of all samples used in this study.
(TIF)

S10 Fig TreeMix model populations splits and pairwise differences between 1c clade species **(A)** TreeMix graph representing splitting of the populations studied, where each population is a 1c clade *Phytophthora* species. Branch lengths are proportional to genetic drift of each population. One migration event, from *P*. *phaseoli* to the common ancestor of *P*. *andina* and *P*. *infestans* is shown. **(B)** Residuals for the TreeMix graph are shown for each pairwise combination of species.
(TIF)

S11 Fig TreeMix model results for numbers and rate of change of migration edges for subpopulations of *Phytophthora infestans* and other 1c clade species. **(A)** Estimation for the optimal number of migration edges with the TreeMix program, developed using OptM. **(B)** Based on the second-order rate of change in likelihood (Δm), 2 is the optimal number of migration edges for a TreeMix model using a dataset comprised of all samples used in this study, with *P*. *infestans* divided into subpopulations and each other species considered as its own population.
(TIF)

S12 Fig TreeMix model populations splits and pairwise differences between subpopulations of *Phytophthora infestans* and other 1c clade species. **(A)** TreeMix graph representing splitting of the populations studied, where each population is a 1c clade *Phytophthora* species except for *P*. *infestans* which is divided further into subpopulations. Branch lengths are proportional to genetic drift of each population. Two migration events, from *P*. *phaseoli* to *P*. *infestans* Historic and from *P*. *ipomoeae* to *P*. *betacei* are shown. **(B)** Residuals for the TreeMix graph are shown for each pairwise combination of species.
(TIF)

S13 Fig Phylogenetic tree derived from mitochondrial genome sequences of each isolate. Each species is highlighted in a different color.
(TIF)

S1 File **S1 Table.** Species of *Phytophthora* from the 1c clade included in this study and their associated metadata; **S2 Table.** Sequencing summary statistics including mean sequencing depth, percent of mapped sequences and average base quality score for sequences and sequencing strategy used for samples in this study; **S3 Table.** Migration rates of 1c clade *Phytophthora* species from pairwise IMa3 runs used to generate data Fig 4. **S4 Table.** Summary of t-tests comparing means for all populations and diversity statistics presented in S4 and S5 Figs.
(DOCX)
