# Supplementary figures and images for: A pangenome analysis reveals the center of origin and evolutionary history of Phytophthora infestans and 1c clade species
Source: PLoS One. 2025 Jan 24;20(1):e0314509. doi: 10.1371/journal.pone.0314509 (PMC11760636; doi:10.1371/journal.pone.0314509)

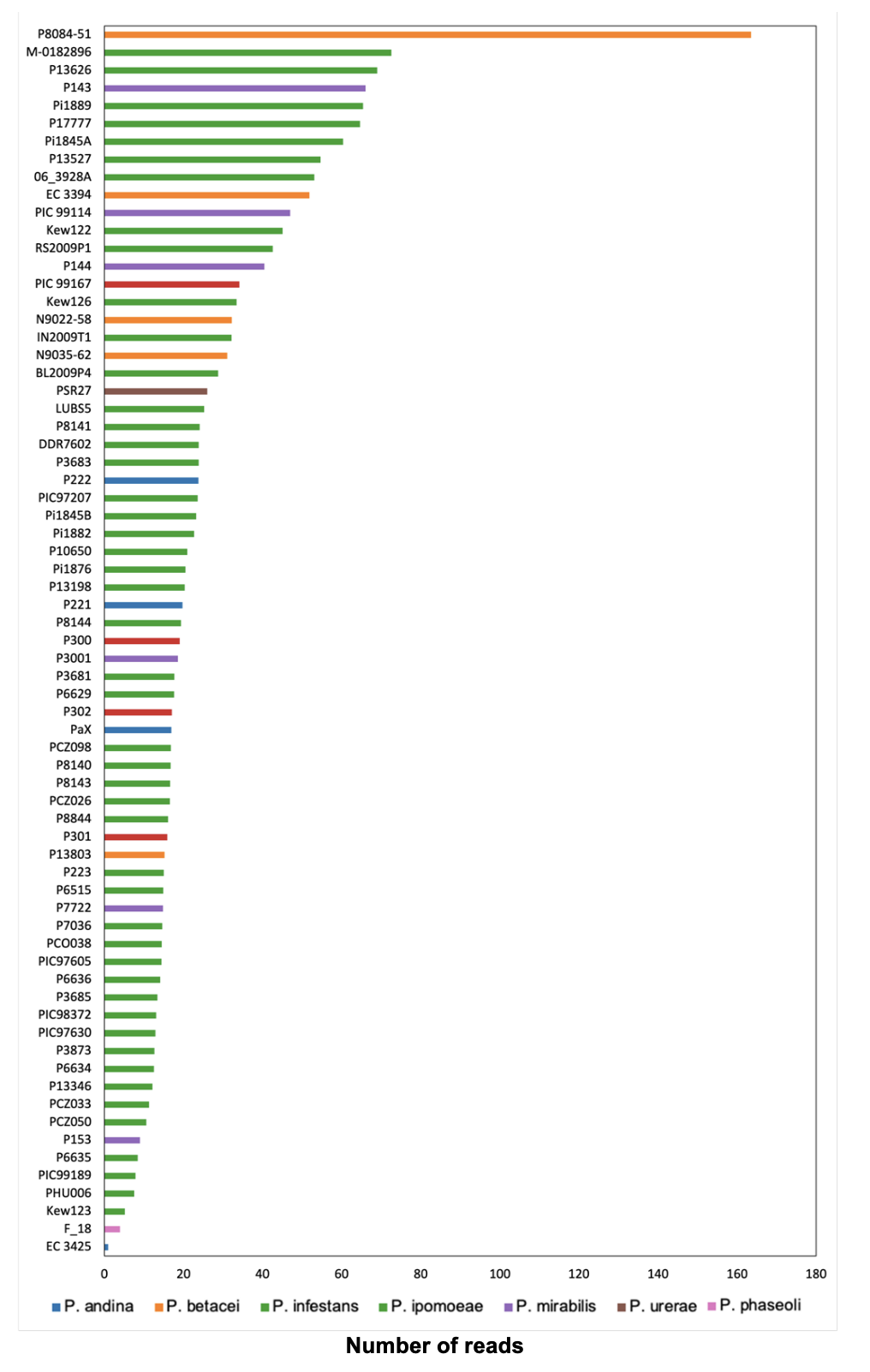

Supplement: S1 Fig — (TIF) [file pone.0314509.s001.tif]

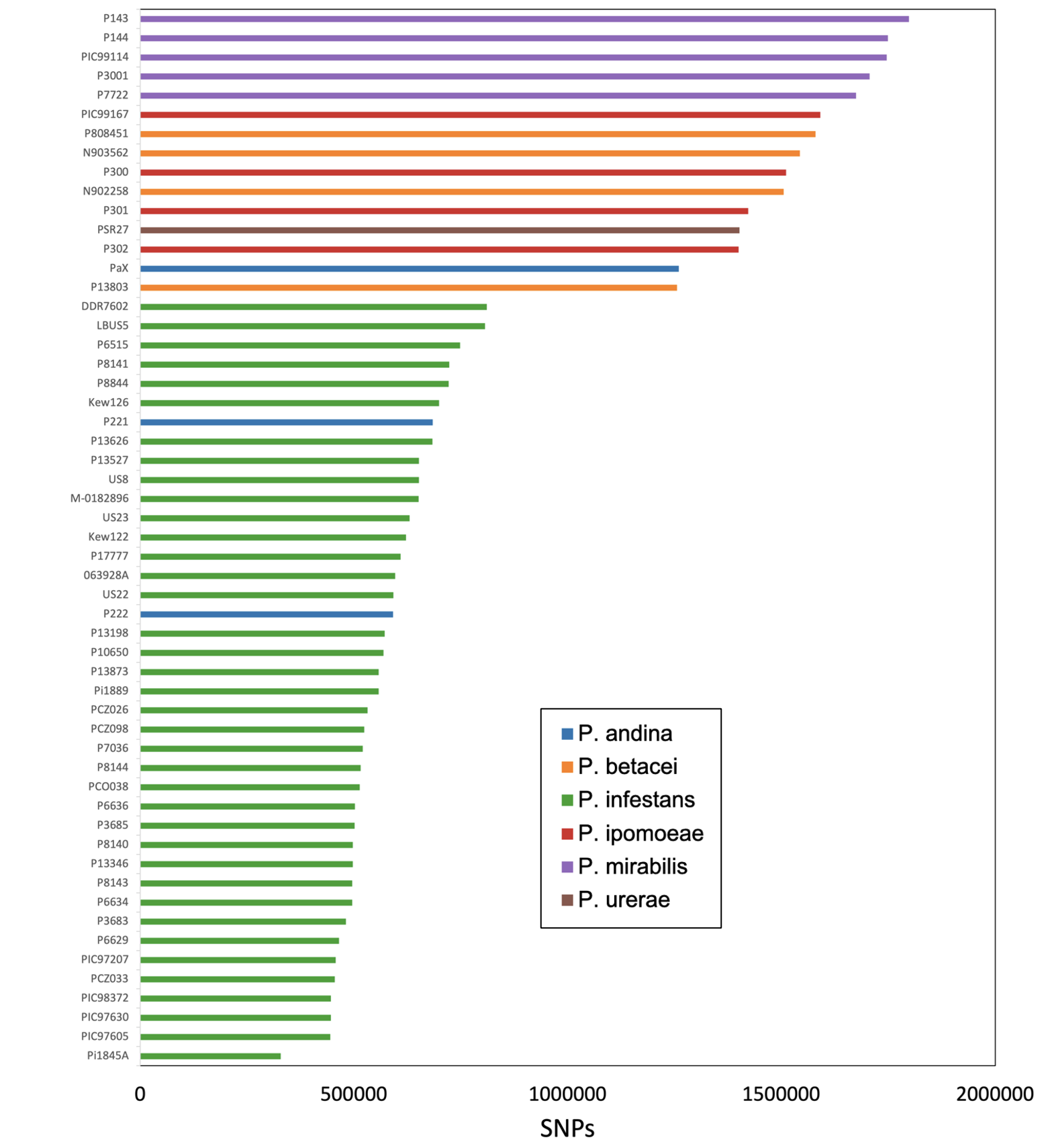

Supplement: S2 Fig — (TIF) [file pone.0314509.s002.tif]

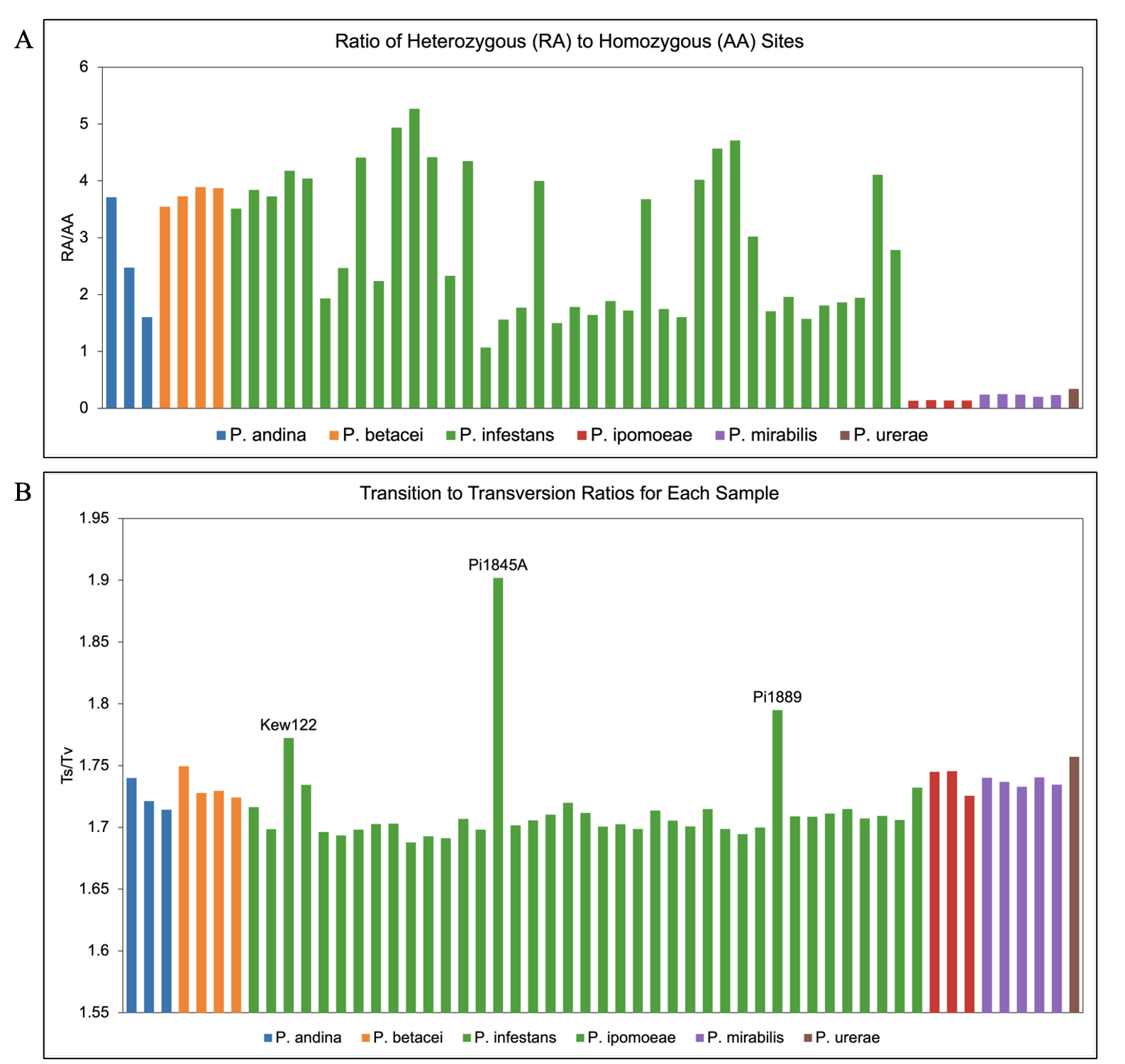

Supplement: S3 Fig — (A) Ratio of heterozygous sites (1 reference allele, 1 alternative allele) compared to homozygous alternate sites (2 alternate alleles). All samples with an average coverage depth at variant sites above 10X are included. (B) Transition to transversion ratio for each sample. All samples with an average coverage depth at variant sites above 10X are included. (TIF) [file pone.0314509.s003.tif]

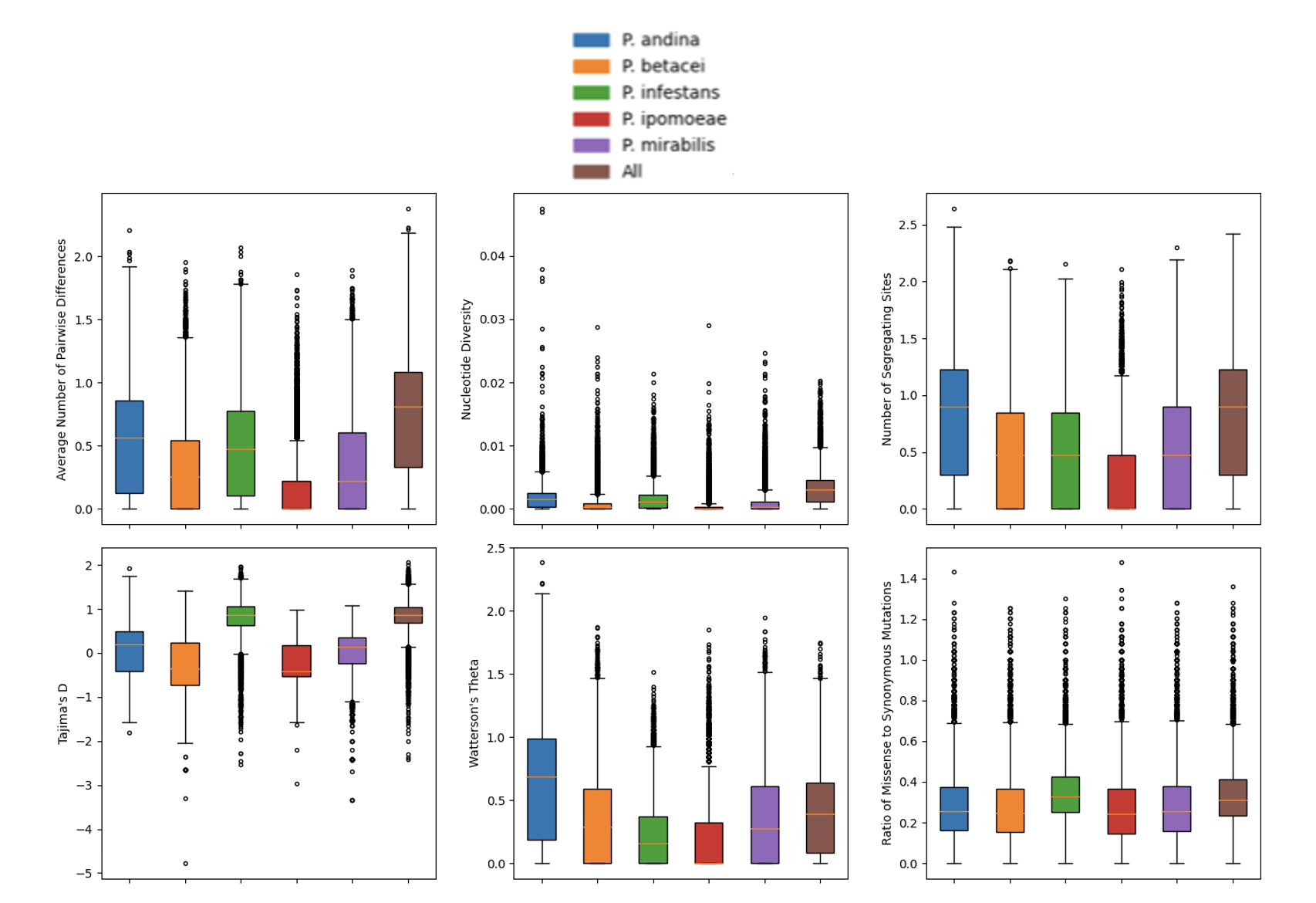

Supplement: S4 Fig — Data is log-transformed to facilitate visualization. (TIF) [file pone.0314509.s004.tif]

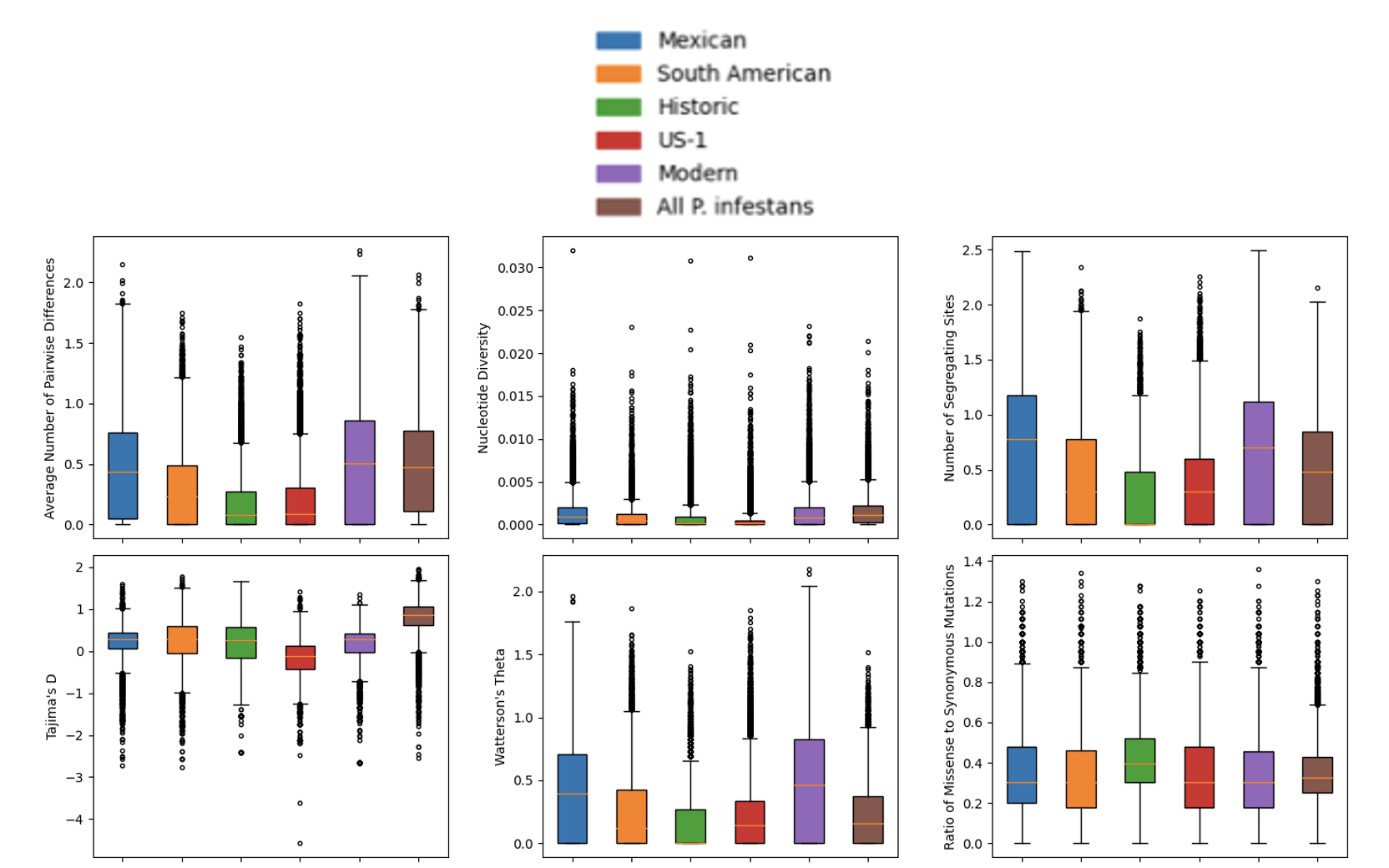

Supplement: S5 Fig — Data is log-transformed to facilitate visualization. (TIF) [file pone.0314509.s005.tif]

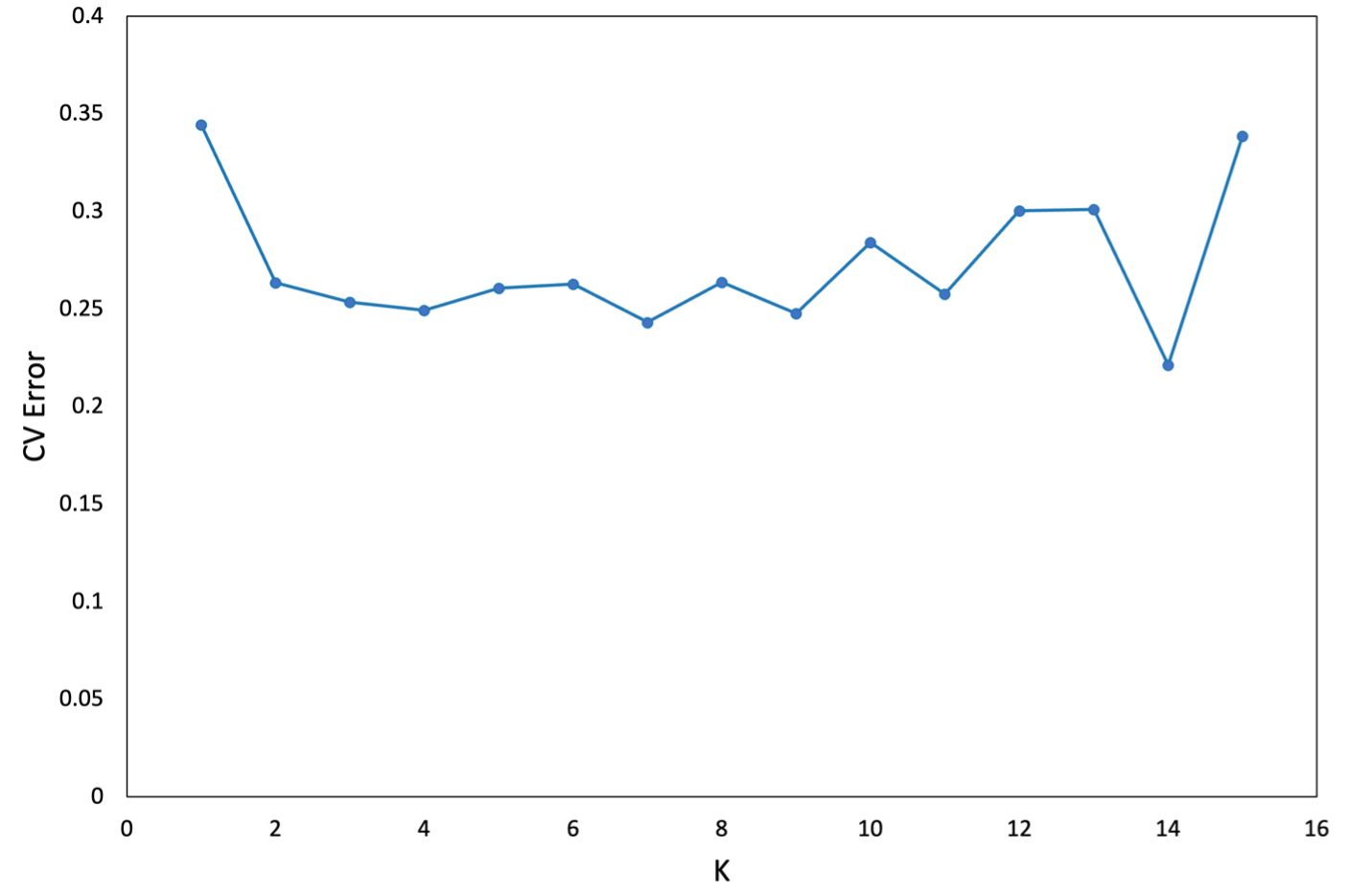

Supplement: S6 Fig — The local minimum around K = 4 shows this to be a reasonable modeling choice. When K> = 7 the variation in the cross-validation increased significantly. K = 7 (the number of recognized species in the dataset) and K = 14 (another local minimum) was also plotted to investigate population groupings within the dataset. (TIF) [file pone.0314509.s006.tif]

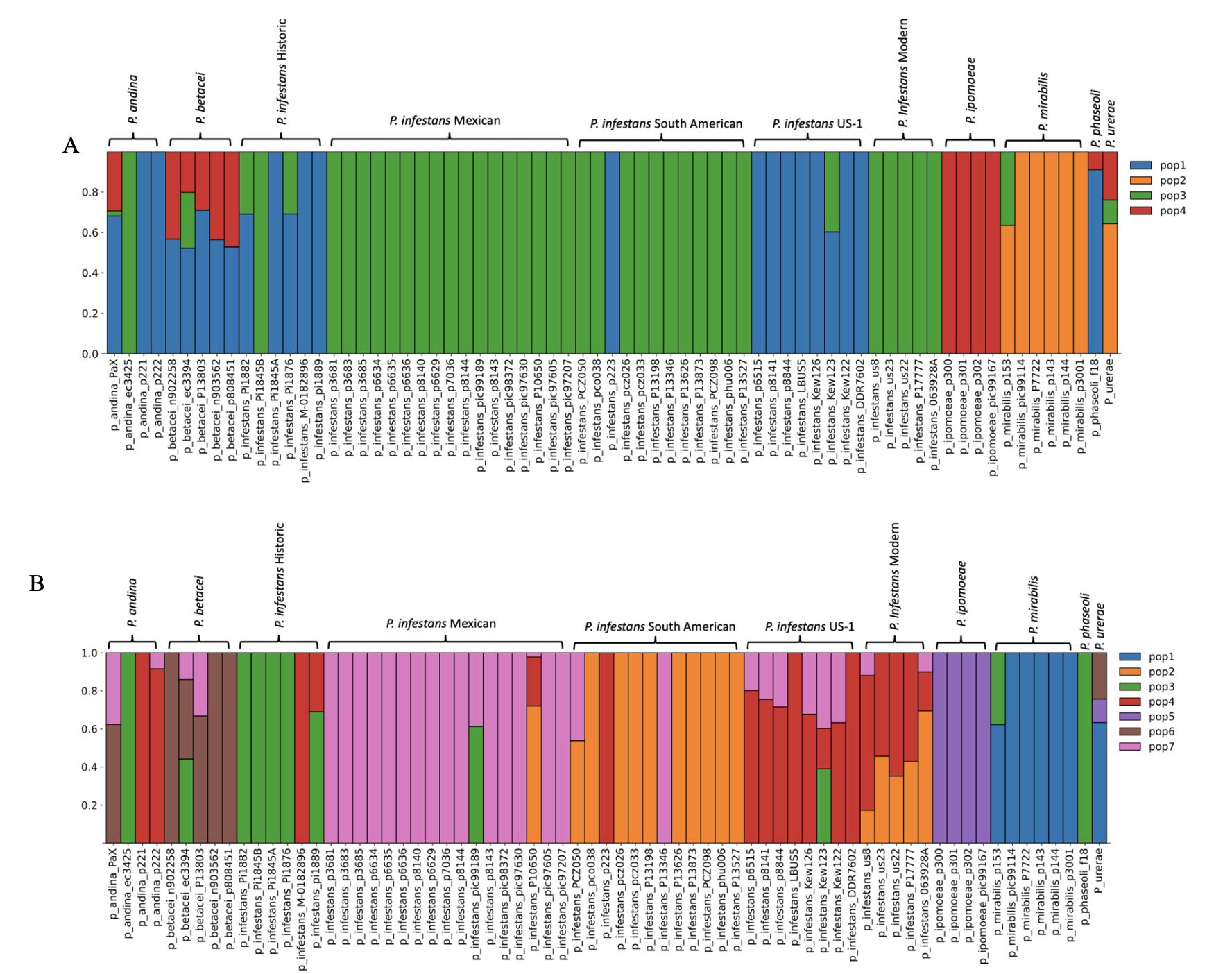

Supplement: S7 Fig — ADMIXTURE plots shown for (A) K = 4 and (B) K = 7. Bars indicate the proportion of genome ancestry from each ancestral population (K). Brackets are shown to represent a simplified breakdown of where each defined population can be found in relation to the ADMIXTURE generated populations. (TIF) [file pone.0314509.s007.tif]

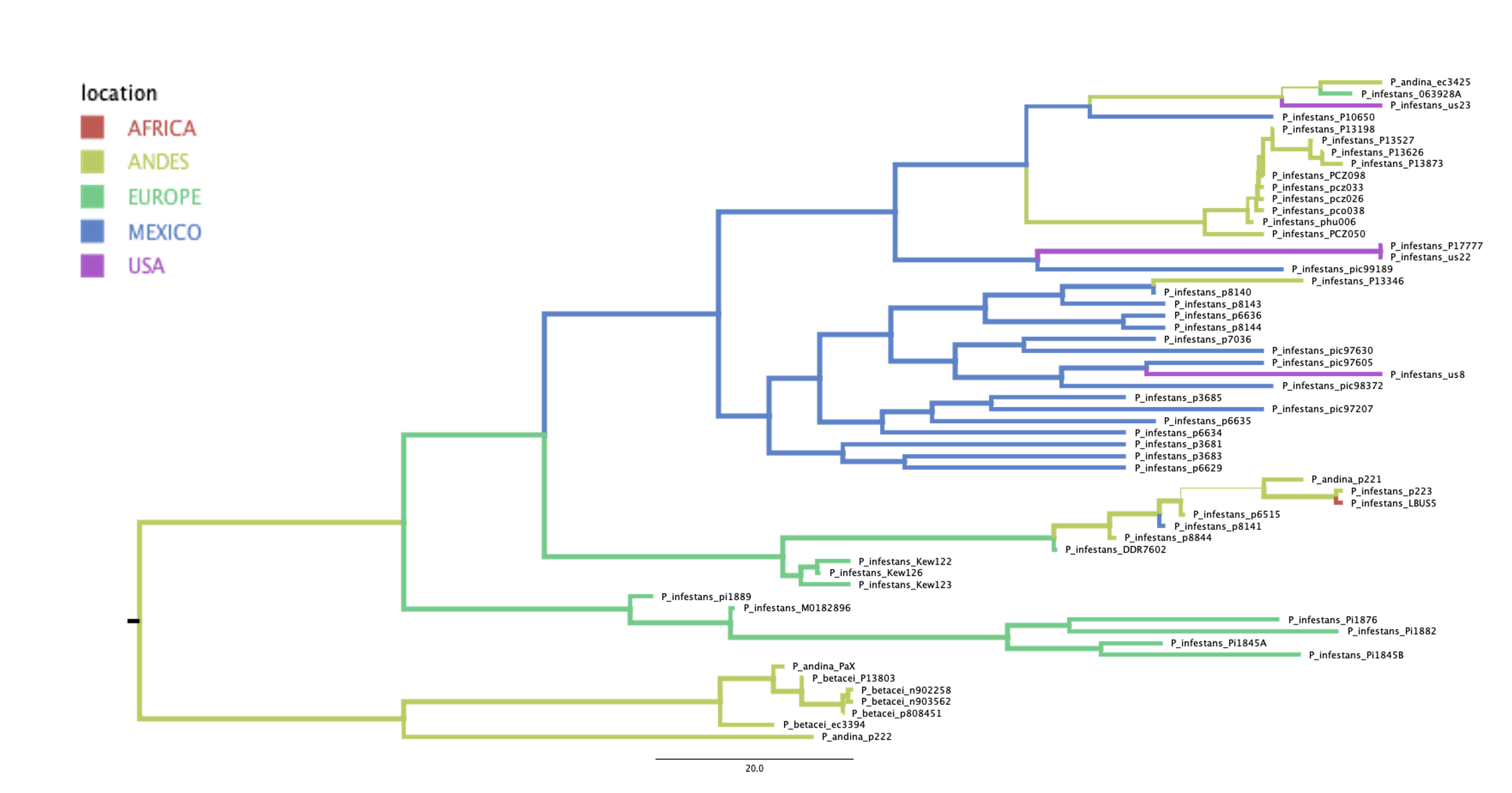

Supplement: S8 Fig — Branches are color coded according to region of origin and branch widths are proportional to posterior support for the branch. The tree is rooted with the P. betacei/P. andina clade as the outgroup. (TIF) [file pone.0314509.s008.tif]

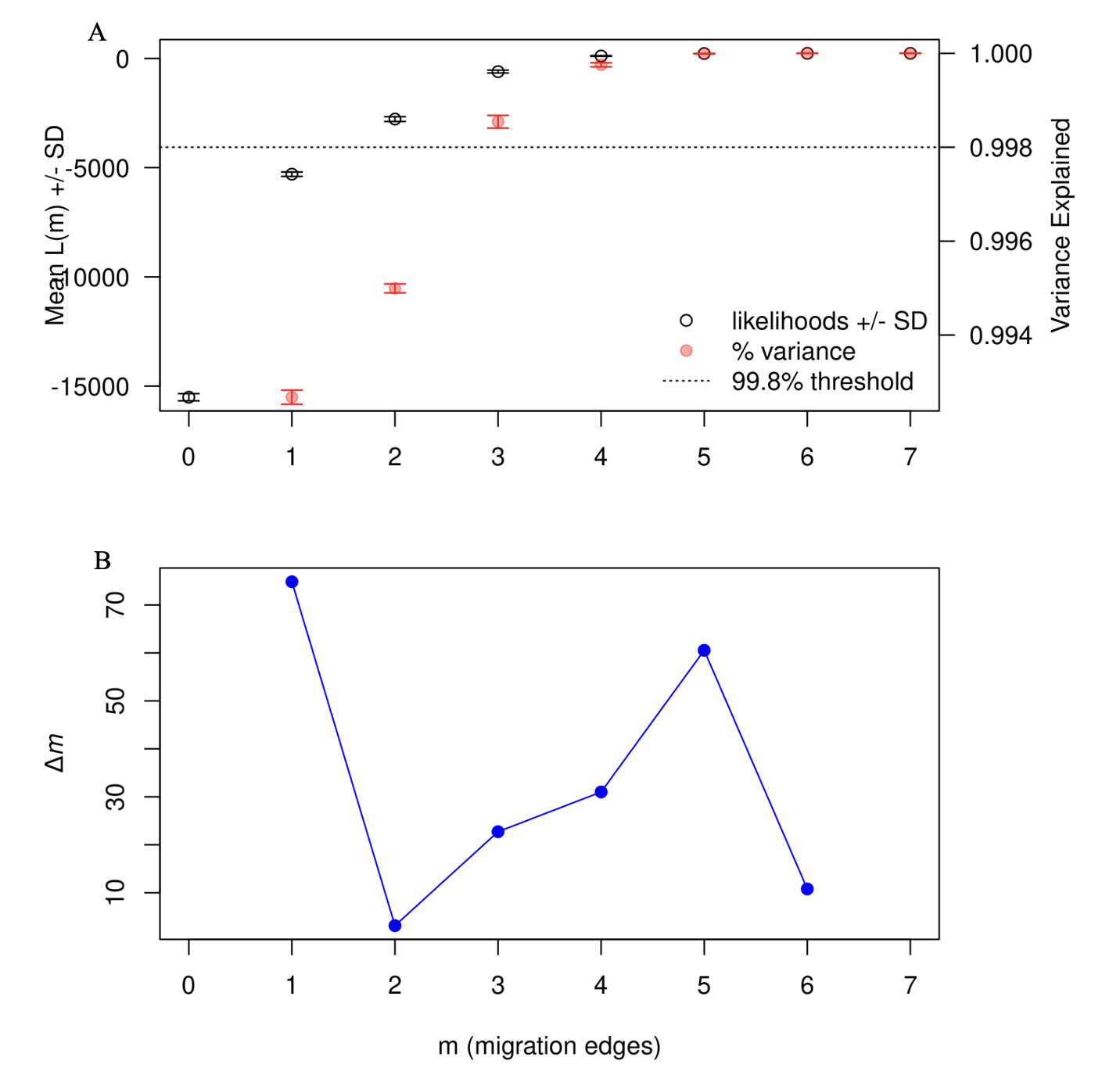

Supplement: S9 Fig — (A) Estimation for the optimal number of migration edges when considering each Phytophthora 1c clade species as a population with the TreeMix program, developed using OptM. (B) Based on the second-order rate of change in likelihood (Δm), 1 is the optimal number of migration edges for a TreeMix model using a dataset comprised of all samples used in this study. (TIF) [file pone.0314509.s009.tif]

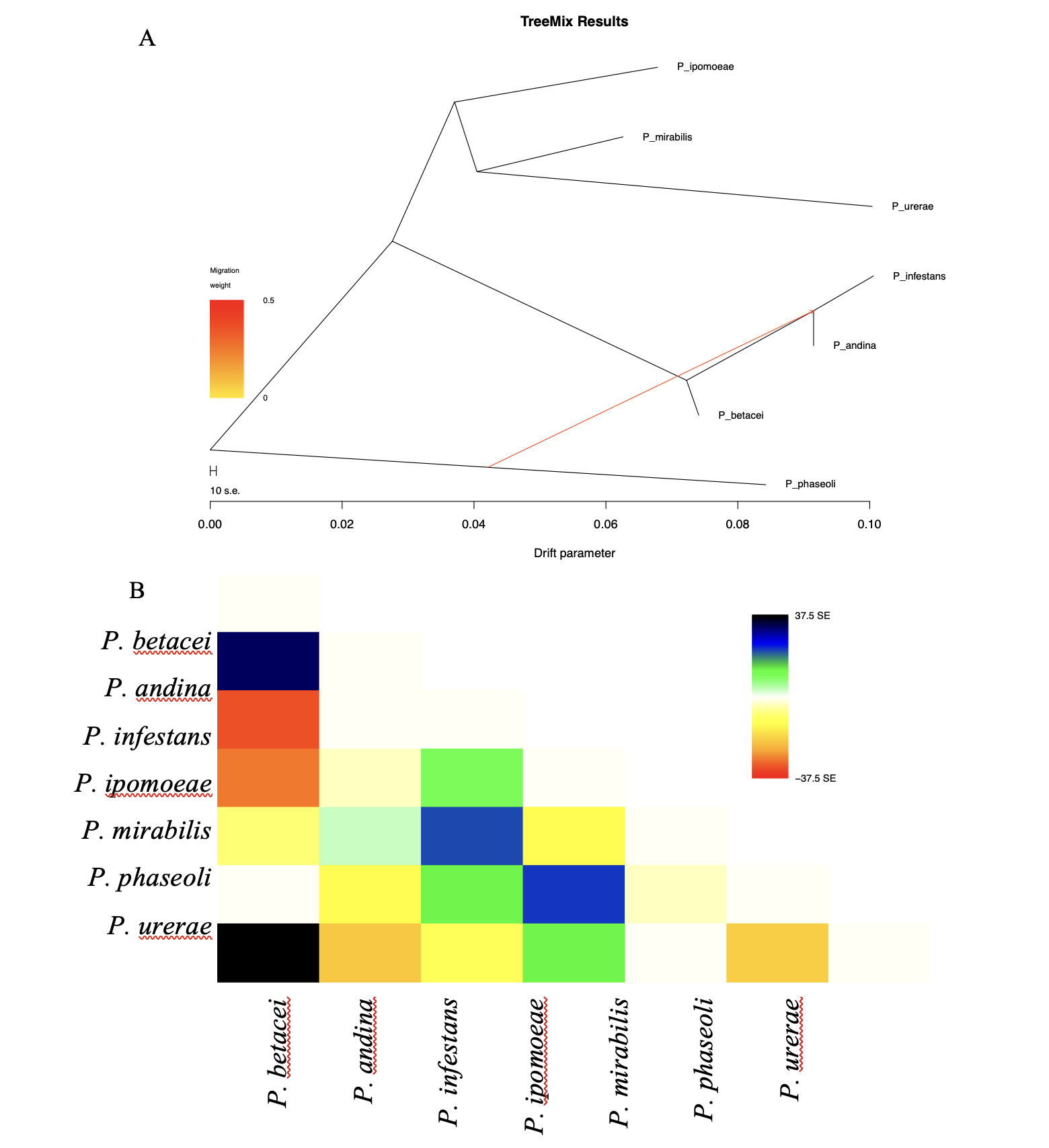

Supplement: S10 Fig — TreeMix model populations splits and pairwise differences between 1c clade species (A) TreeMix graph representing splitting of the populations studied, where each population is a 1c clade Phytophthora species. Branch lengths are proportional to genetic drift of each population. One migration event, from P. phaseoli to the common ancestor of P. andina and P. infestans is shown. (B) Residuals for the TreeMix graph are shown for each pairwise combination of species. (TIF) [file pone.0314509.s010.tif]

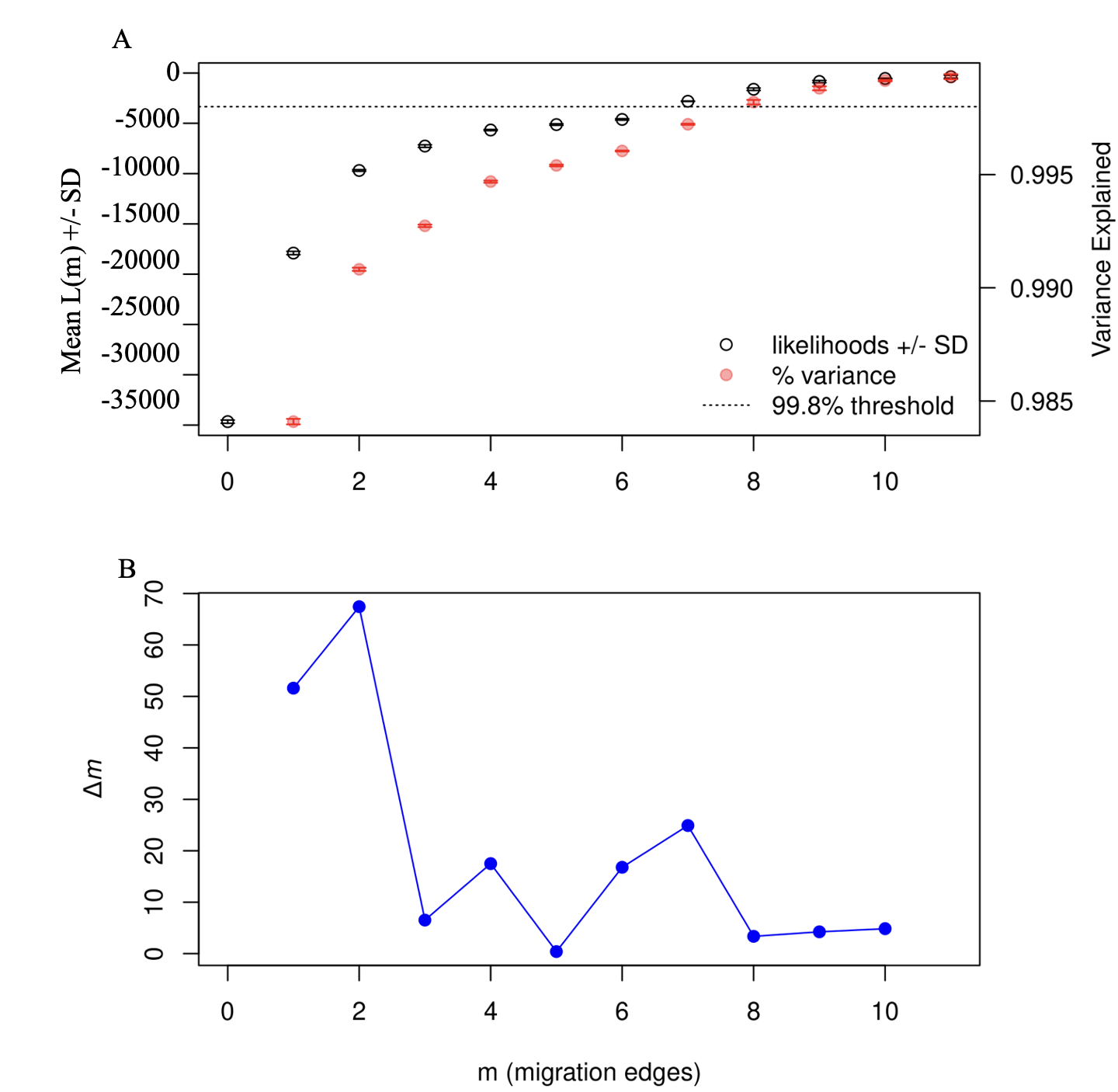

Supplement: S11 Fig — (A) Estimation for the optimal number of migration edges with the TreeMix program, developed using OptM. (B) Based on the second-order rate of change in likelihood (Δm), 2 is the optimal number of migration edges for a TreeMix model using a dataset comprised of all samples used in this study, with P. infestans divided into subpopulations and each other species considered as its own population. (TIF) [file pone.0314509.s011.tif]

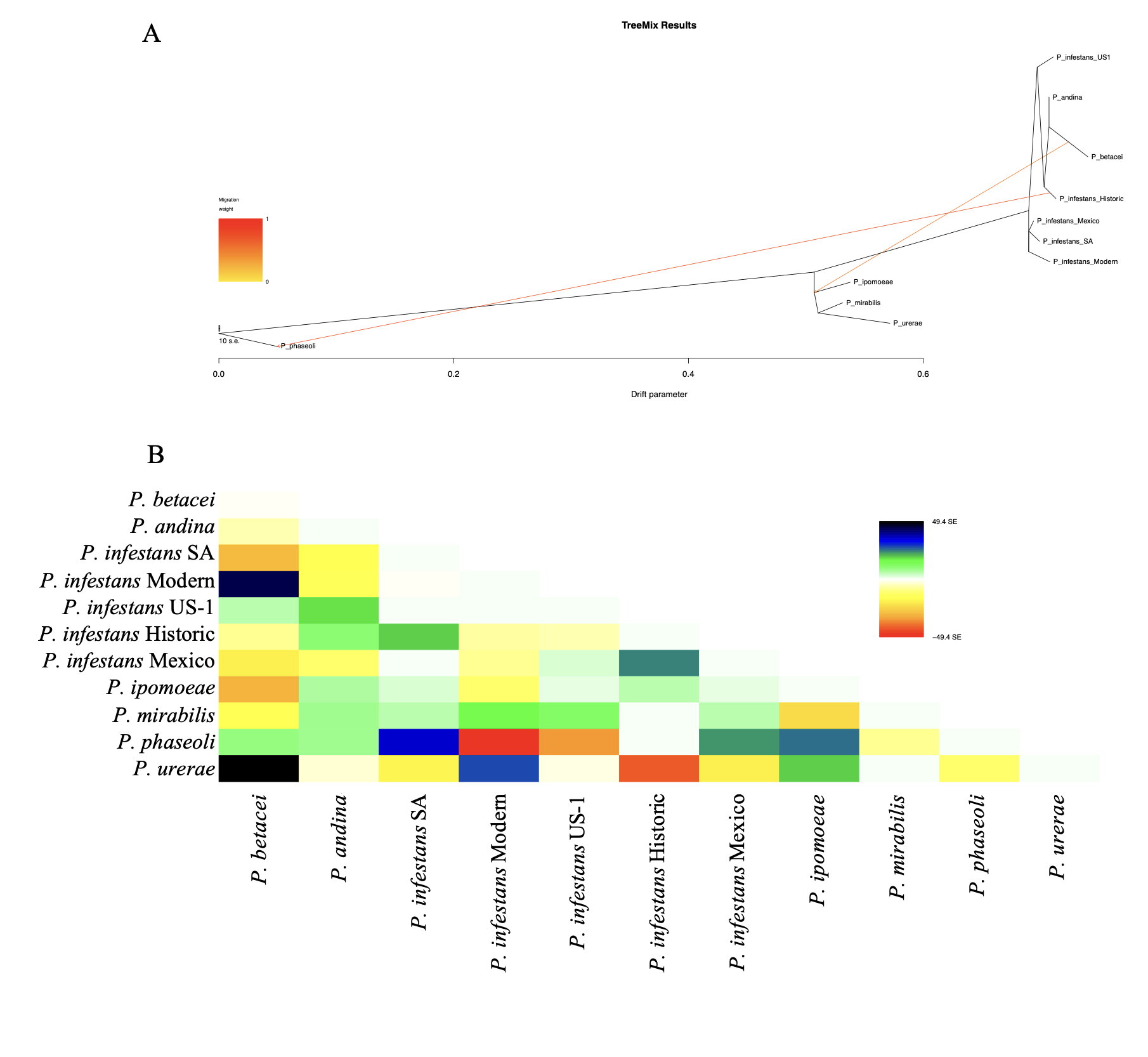

Supplement: S12 Fig — (A) TreeMix graph representing splitting of the populations studied, where each population is a 1c clade Phytophthora species except for P. infestans which is divided further into subpopulations. Branch lengths are proportional to genetic drift of each population. Two migration events, from P. phaseoli to P. infestans Historic and from P. ipomoeae to P. betacei are shown. (B) Residuals for the TreeMix graph are shown for each pairwise combination of species. (TIF) [file pone.0314509.s012.tif]

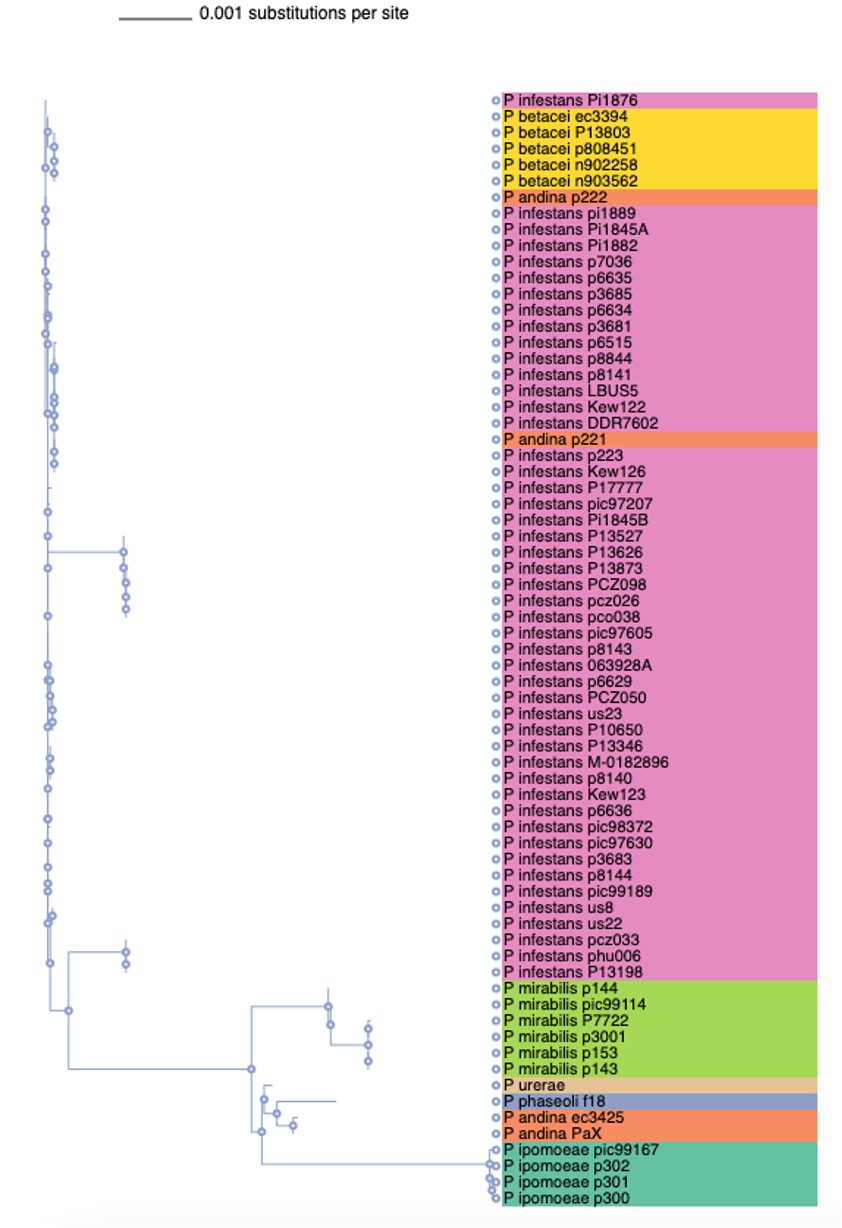

Supplement: S13 Fig — Each species is highlighted in a different color. (TIF) [file pone.0314509.s013.tif]
