# Supplementary material for: A pangenome analysis reveals the center of origin and evolutionary history of Phytophthora infestans and 1c clade species
Source: PLoS One. 2025 Jan 24;20(1):e0314509. doi: 10.1371/journal.pone.0314509 (PMC11760636; doi:10.1371/journal.pone.0314509)
Supplement: S1 File — S1 Table. Species of Phytophthora from the 1c clade included in this study and their associated metadata; S2 Table. Sequencing summary statistics including mean sequencing depth, percent of mapped sequences and average base quality score for sequences and sequencing strategy used for samples in this study; S3 Table. Migration rates of 1c clade Phytophthora species from pairwise IMa3 runs used to generate data Fig 4. S4 Table. Summary of t-tests comparing means for all populations and diversity statistics presented in S4 and S5 Figs. (DOCX) [file pone.0314509.s014.docx]

Supplementary Materials for

A pangenome analysis reveals the center of origin and evolutionary history of *Phytophthora infestans* and 1c clade species.

Allison L. Coomber, Amanda C. Saville, Ignazio Carbone, Michael Martin, Vanessa C. Bieker, Jean Beagle Ristaino

Corresponding author: Jean_ristaino@ncsu.edu

**S1 Table.** **Species of *Phytophthora* from the 1c clade included in this study and their associated metadata.** Summary of samples included in the study along with which populations they were assigned during population level analysis. Each population comprised one 1c clade species, except for *P. infestans* which was divided into smaller subspecies level populations.

| **Population^a^** | **Species** | **Isolate** | **GT^b^** | **MT^c^** | **Host** | **Year** | **Location** | **Collector** | **Sequence Reference** |
| --- | --- | --- | --- | --- | --- | --- | --- | --- | --- |
| *P. andina* | *P. andina* | EC 3425 | EC-2.1 | Ic | *S. brevifolium* | 2001 | Ecuador | W. Flier | ENA PRJNA52431 |
| *P. andina* | *P. andina* | P221 |  | Ia | *S. quitoense* | 2001 | Ecuador | G. Chacon | SRR26725399^d^ |
| *P. andina* | *P. andina* | P222 |  | Ia | *S. ochranthum* | 2005 | Ecuador | G. Chacon | SRR26725398^d^ |
| *P. andina* | *P. andina* | PaX |  | Ic | *Solanum spp.* |  |  |  | [25] |
| *P. betacei* | *P. betacei* | EC 3394 | EC-3 | Ia | *S. betaceum* | 2001 | Ecuador | G. Forbes | [25] |
| *P. betacei* | *P. betacei* | N9022-58 | EC-3 | Ia | *S. betaceum* | 2009 | Colombia | S. Restrepo | SRR26725390^d^ |
| *P. betacei* | *P. betacei* | P8084-51 | EC-3 | Ia | *S. betaceum* | 2008 | Colombia | S. Restrepo | [31], SRR26725388^d^ |
| *P. betacei* | *P. betacei* | N9035-62 |  |  | *S. betaceum* | 2009 | Colombia | S. Restrepo | SRR26725389^d^ |
| *P. betacei* | *P. betacei* | P13803 | EC-3 | HERB-1 | *S. betaceum* | 2004 | Ecuador |  | [25] |
| *P. infestans* Historic | *P. infestans* | Pi1889 | FAM-1 | HERB-1 | *S. tuberosum* | 1889 | Germany | P. Hennings | [62] |
| *P. infestans* Historic | *P. infestans* | M-0182896 |  | HERB-1 | *S. tuberosum* | 1877 | Germany |  | [63] |
| *P. infestans* Historic | *P. infestans* | Pi1845A |  | HERB-1 | *S. tuberosum* | 1845 | Belgium | J. B. Desmazieres | [62] |
| *P. infestans* Historic | *P. infestans* | Pi1876 | FAM-1 | HERB-1 | *S. tuberosum* | 1876 | Denmark | E. Rostrup | [62] |
| *P. infestans* Historic | *P. infestans* | Pi1882 | FAM-1 | HERB-1 | *S. tuberosum* | 1882 | Sweden | J. Eriksson | [62] |
| *P. infestans* Historic | *P. infestans* | Pi1845B |  | HERB-1 | *S. tuberosum* | 1845 | Britain | J. Lindley | [62] |
| *P. infestans* Mexico | *P. infestans* | PIC97207 |  | Ia | *S. tuberosum* | 1997 | Mexico | W. Fry | [25] |
| *P. infestans* Mexico | *P. infestans* | PIC99189* |  | Ia | *S. stoloniferum* | 1999 | Mexico | W. Fry | [64] |
| *P. infestans* Mexico | *P. infestans* | PIC97605 |  | Ia | *S. tuberosum* | 1997 | Mexico | W. Fry | [25] |
| *P. infestans* Mexico | *P. infestans* | PIC97630 |  | Ia | *S. tuberosum* | 1997 | Mexico | W. Fry | [25] |
| *P. infestans* Mexico | *P. infestans* | PIC98372 |  | Ia | *S. demissum* | 1998 | Mexico | W. Fry | [25] |
| *P. infestans* Mexico | *P. infestans* | P3683 |  | Ia | *S. stoloniferum* | 1983 | Mexico | W. Fry | [25] |
| *P. infestans* Mexico | *P. infestans* | P8144 |  | Ia | *S. tuberosum* | 1987 | Mexico | W. Fry | [25] |
| *P. infestans* Mexico | *P. infestans* | P8140 |  | HERB-1 | *S. tuberosum* | 1986 | Mexico | W. Fry | [25] |
| *P. infestans* Mexico | *P. infestans* | P8143 |  | Ia | *S. tuberosum* | 1987 | Mexico | W. Fry | [25] |
| *P. infestans* Mexico | *P. infestans* | P7036 |  | HERB-1 | *S. tuberosum* | 1986 | Mexico | W. Fry | [25] |
| *P. infestans* Mexico | *P. infestans* | P6636 |  | Ia | *S. tuberosum* | 1987 | Mexico | W. Fry | [25] |
| *P. infestans* Mexico | *P. infestans* | P6629 |  | Ia | *S. tuberosum* | 1983 | Mexico | W. Fry | [25] |
| *P. infestans* Mexico | *P. infestans* | P3685 |  | Ia | *S. tuberosum* | 1983 | Mexico | W. Fry | [25] |
| *P. infestans* Mexico | *P. infestans* | P6634 |  | Ia | *S. tuberosum* | 1983 | Mexico | W. Fry | [25] |
| *P. infestans* Mexico | *P. infestans* | P3681 |  | Ia | *S. tuberosum* | 1983 | Mexico | W. Fry | [25] |
| *P. infestans* Mexico | *P. infestans* | P10650* |  | Ia | *S. tuberosum* | 1998 | Mexico | W. Fry | [25, 63] |
| *P. infestans* Mexico | *P. infestans* | P6635 |  | Ia | *S. demissum* | 1986 | Mexico | W. Fry | [25] |
| *P. infestans* Modern | *P. infestans* | IN2009T1* | US-22 | Ia | *S. lycopersicum* | 2009 | PA, USA | J. Ristaino | [62] |
| *P. infestans* Modern | *P. infestans* | BL2009P4* | US-23 | Ia | *S. tuberosum* | 2009 | PA, USA | J. Ristaino | [62] |
| *P. infestans* Modern | *P. infestans* | RS2009P1* | US-8 | Ia | *S. tuberosum* | 2009 | PA, USA | J. Ristaino | [62],  SRR26725392 ^e^ |
| *P. infestans* Modern | *P. infestans* | P17777* | US-22 | Ia | *S. lycopersicum* | 2009 | USA | J. Ristaino | [63] |
| *P. infestans* Modern | *P. infestans* | 06_3928A* | EU-13 | Ia | *S. tuberosum* | 2006 | Britain | D. Cooke | [65] |
| *P. infestans* SA | *P. infestans* | P223* |  |  |  | 1997 | Ecuador | G. Forbes | SRR26725387^d^ |
| *P. infestans* SA | *P. infestans* | PCO038 | EC-1 | IIa | *S. tuberosum* | 1997 | Peru | G. Forbes | [25] |
| *P. infestans* SA | *P. infestans* | PHU006 | EC-1 | IIa | *S. tuberosum* | 1996 | Peru | G. Forbes | [25] |
| *P. infestans* SA | *P. infestans* | PCZ026 | PE-6 | IIa | *S. tuberosum* | 1997 | Peru | G. Forbes | [25] |
| *P. infestans* SA | *P. infestans* | PCZ033 | EC-1.1 | IIa | *S. tuberosum* | 1997 | Peru | G. Forbes | [25] |
| *P. infestans* SA | *P. infestans* | P13198 | EC-1 | IIa | *S. tuquerrense* | 1998 | Ecuador | G. Forbes | [25] |
| *P. infestans* SA | *P. infestans* | P13626 | EC-1 | IIa | *S. tuberosum* | 2003 | Ecuador | G. Forbes | [25] |
| *P. infestans* SA | *P. infestans* | P13527 |  | IIa | *S. andreanum* | 2002 | Ecuador | G. Forbes | [63] |
| *P. infestans* SA | *P. infestans* | PCZ098 | EC-1.3 | IIa | *S. tuberosum* | 1997 | Peru | G. Forbes | [25] |
| *P. infestans* SA | *P. infestans* | P13873 | EC-1 | IIa | *S. tuberosum* | 2005 | Ecuador | G. Forbes | [25] |
| *P. infestans* SA | *P. infestans* | P13346* | EC-1 | HERB-1 | *S. colombianum* | 2001 | Ecuador | G. Forbes | [25] |
| *P. infestans* SA | *P. infestans* | PCZ050 | PE-3 | Ia | *S. tuberosum* | 1997 | Peru | G. Forbes | [25] |
| *P. infestans* US-1 | *P. infestans* | P6515 | US-1 | Ib | *S. tuberosum* | 1989 | Peru | G. Forbes | [25] |
| *P. infestans* US-1 | *P. infestans* | LUBS5 | US-1 | Ib | *Petunia hybrida* | 2005 | South Africa |  | [63] |
| *P. infestans* US-1 | *P. infestans* | DDR7602 | US-1 | Ib | *S. tuberosum* | 1976 | Germany |  | [63] |
| *P. infestans* US-1 | *P. infestans* | P8141 | US-1 | Ib | *S. tuberosum* | 1987 | Mexico | W. Fry | [25] |
| *P. infestans* US-1 | *P. infestans* | Kew126 | US-1 | Ib | *S. tuberosum* | 1952 | Britain | J. H. H. | [25] |
| *P. infestans* US-1 | *P. infestans* | P8844 | US-1 | Ib | *S. tuberosum* | 1982 | Peru | H. Hohl | [25] |
| *P. infestans* US-1 | *P. infestans* | Kew122 | US-1 | Ib | *S. tuberosum* | 1955 | Britain | R. Clark | [25] |
| *P. infestans* US-1 | *P. infestans* | Kew123 | US-1 | Ib | *S. tuberosum* | 1955 | Ireland | R. Dennis | [25] |
| *P. ipomoeae* | *P. ipomoeae* | P301 | NA | NA | *Ipomoea longipedunculata* | 1999 | Mexico | W. Flier | SRR26725385^d^ |
| *P. ipomoeae* | *P. ipomoeae* | PIC 99167 | NA | NA | *Ipomoea longipedunculata* | 1999 | Mexico | W. Flier | SRR26725393^e^ [63,64] |
| *P. ipomoeae* | *P. ipomoeae* | P300 | NA | NA | *Ipomoea longipedunculata* | 2005 | Mexico | W. Flier | SRR26725386^d^ |
| *P. ipomoeae* | *P. ipomoeae* | P302 | NA | NA | *Ipomoea longipedunculata* | 2005 | Mexico | W. Flier | SRR26725384^d^ |
| *P. mirabilis* | *P. mirabilis* | P153 | NA | NA | *Mirabilis jalapa* | 2004 | Mexico | M. Gallegly | This study ^d^ |
| *P. mirabilis* | *P. mirabilis* | PIC 99114 | NA | NA | *Mirabilis jalapa* | 1999 | Mexico |  | [63,64] |
| *P. mirabilis* | *P. mirabilis* | P3001 | NA | NA | *Mirabilis jalapa* | 2004 | Mexico | M. Gallegly | SRR26725396^d^ |
| *P. mirabilis* | *P. mirabilis* | P143 | NA | NA | *Mirabilis jalapa* | 2004 | Mexico | M. Gallegly | SRR26725383^d^ |
| *P. mirabilis* | *P. mirabilis* | P144 | NA | NA | *Mirabilis jalapa* | 2004 | Mexico | M. Gallegly | SRR26725397, SRR26725391^de^ |
| *P. mirabilis* | *P. mirabilis* | P7722 | NA | NA | *S. lycopersicum* | 1992 | United States |  | [63] |
| *P. phaseoli* | *P. phaseoli* | F_18 | NA | NA | *P. lunatus* | 2000 | Delaware | T. Evans | ENA PRJNA52439, SRR26725394 ^e^ |
| *P. urerae* | *P. urerae* | PSR27 | NA | NA | *Urera laciniata* |  | CIP, Peru | S. Gamboa | SRR26725395^d^ |

^a^Each population was comprised of one 1c clade species, with the exception for *P. infestans* which was divided into five subspecies level populations. Note isolates *P andina* 221 and 222 were reclassified to *P. infestans* in this work and NCBI SRA list these as *P. infestans*

^b^ SSR genotype

^c^ mitochondrial DNA haplotype

^d^ Sequencing was done in a paired-end format using an Illumina NovaSeq platform.

^e^ Sequencing was done with the Oxford Nanopore MinION platform

*Sample is not included in *P. infestans* population IMa3 analysis based on phylogenetic and network analysis due to not grouping with the rest of the population in SplitsTree and RaXML analyses.

**S2 Table.** **Sequencing summary statistics including mean sequencing depth, percent of mapped sequences and average base quality score for sequences** **and sequencing strategy used for samples in this study**. The coverage statistics are generated from sequence data obtained from PacBio (P), MinION (M), or Illumina (I) projects (see Table S1 references).

| **Population** | **Species** | **Sample ID** | **Mean Coverage Depth^b^** | **Percent Mapped^c^** | **Average Quality Score^d^** | **Sequencing Strategy**^e^ |
| --- | --- | --- | --- | --- | --- | --- |
| *P. andina* | *P. andina* | EC 3425 | 0.93 | 87.58% | 35.8 | I |
| *P. andina* ^a^ | *P. andina* | P221 | 19.74 | 98.75% | 36.3 | I |
| *P. andina* ^a^ | *P. andina* | P222 | 23.77 | 99.05% | 36.3 | I |
| *P. andina* | *P. andina* | PaX | 16.93 | 95.07% | 35.3 | I |
| *P. betacei* | *P. betacei* | EC 3394 | 51.85 | 95.65% | 36.0 | I |
| *P. betacei* | *P. betacei* | N9022-58 | 32.19 | 97.44% | 35.6 | I |
| *P. betacei* | *P. betacei* | N9035-62 | 31.05 | 97.34% | 36.1 | I |
| *P. betacei* | *P. betacei* | P13803 | 15.15 | 96.00% | 35.1 | I |
| *P. betacei* | *P. betacei* | P8084-51 | 163.53 | 97.80% | 19.8 | P, I |
| *P. infestans* Historic | *P. infestans* | M-0182896 | 72.54 | 22.05% | 36.3 | I |
| *P. infestans* Historic | *P. infestans* | Pi1845A | 60.35 | 14.91% | 36.4 | I |
| *P. infestans* Historic | *P. infestans* | Pi1845B | 23.20 | 17.63% | 35.0 | I |
| *P. infestans* Historic | *P. infestans* | Pi1876 | 20.49 | 12.30% | 36.7 | I |
| *P. infestans* Historic | *P. infestans* | Pi1882 | 22.69 | 11.36% | 35.6 | I |
| *P. infestans* Historic | *P. infestans* | Pi1889 | 65.39 | 20.80% | 35.8 | I |
| *P. infestans* Mexico | *P. infestans* | P10650 | 20.91 | 98.54% | 35.2 | I |
| *P. infestans* Mexico | *P. infestans* | P3683 | 23.80 | 98.42% | 35.0 | I |
| *P. infestans* Mexico | *P. infestans* | P3685 | 13.40 | 98.73% | 34.4 | I |
| *P. infestans* Mexico | *P. infestans* | P3873 | 12.62 | 98.75% | 34.4 | I |
| *P. infestans* Mexico | *P. infestans* | P6629 | 17.61 | 97.71% | 35.5 | I |
| *P. infestans* Mexico | *P. infestans* | P6634 | 12.50 | 97.75% | 34.9 | I |
| *P. infestans* Mexico | *P. infestans* | P6635 | 8.39 | 60.28% | 34.9 | I |
| *P. infestans* Mexico | *P. infestans* | P6636 | 14.08 | 98.19% | 34.6 | I |
| *P. infestans* Mexico | *P. infestans* | P7036 | 14.62 | 97.37% | 34.9 | I |
| *P. infestans* Mexico | *P. infestans* | P8140 | 16.72 | 98.17% | 34.6 | I |
| *P. infestans* Mexico | *P. infestans* | P8143 | 16.57 | 98.16% | 35.3 | I |
| *P. infestans* Mexico | *P. infestans* | P8144 | 19.33 | 97.95% | 34.7 | I |
| *P. infestans* Mexico | *P. infestans* | PIC97207 | 23.54 | 86.68% | 37.1 | I |
| *P. infestans* Mexico | *P. infestans* | PIC97605 | 14.42 | 98.41% | 37.0 | I |
| *P. infestans* Mexico | *P. infestans* | PIC97630 | 12.87 | 98.18% | 37.1 | I |
| *P. infestans* Mexico | *P. infestans* | PIC98372 | 13.06 | 96.79% | 36.6 | I |
| *P. infestans* Mexico | *P. infestans* | PIC99189 | 7.82 | 90.33% | 36.1 | I |
| *P. infestans* Modern | *P. infestans* | 06_3928A | 53.03 | 98.73% | 28.5 | I |
| *P. infestans* Modern | *P. infestans* | BL2009P4 | 28.71 | 99.25% | 36.5 | I |
| *P. infestans* Modern | *P. infestans* | IN2009T1 | 32.10 | 99.13% | 36.6 | I |
| *P. infestans* Modern | *P. infestans* | P223 | 14.98 | 98.45% | 36.3 | I |
| *P. infestans* Modern | *P. infestans* | RS2009P1 | 42.55 | 99.26% | 33.4 | M, I |
| *P. infestans* SA | *P. infestans* | P13198 | 20.28 | 98.31% | 35.1 | I |
| *P. infestans* SA | *P. infestans* | P13346 | 12.14 | 98.29% | 34.4 | I |
| *P. infestans* SA | *P. infestans* | P13527 | 54.64 | 98.60% | 37.8 | I |
| *P. infestans* SA | *P. infestans* | P13626 | 68.94 | 98.64% | 37.5 | I |
| *P. infestans* SA | *P. infestans* | P17777 | 64.63 | 99.26% | 31.7 | I |
| *P. infestans* SA | *P. infestans* | P3681 | 17.69 | 98.83% | 34.3 | I |
| *P. infestans* SA | *P. infestans* | PCO038 | 14.47 | 98.47% | 37.0 | I |
| *P. infestans* SA | *P. infestans* | PCZ026 | 16.52 | 98.59% | 36.9 | I |
| *P. infestans* SA | *P. infestans* | PCZ033 | 11.27 | 98.54% | 37.0 | I |
| *P. infestans* SA | *P. infestans* | PCZ050 | 10.59 | 98.56% | 37.0 | I |
| *P. infestans* SA | *P. infestans* | PCZ098 | 16.78 | 98.42% | 37.0 | I |
| *P. infestans* SA | *P. infestans* | PHU006 | 7.51 | 98.40% | 34.6 | I |
| *P. infestans* US-1 | *P. infestans* | DDR7602 | 23.82 | 99.05% | 37.6 | I |
| *P. infestans* US-1 | *P. infestans* | Kew122 | 45.04 | 19.90% | 36.4 | I |
| *P. infestans* US-1 | *P. infestans* | Kew123 | 5.17 | 28.30% | 36.3 | I |
| *P. infestans* US-1 | *P. infestans* | Kew126 | 33.43 | 20.52% | 36.6 | I |
| *P. infestans* US-1 | *P. infestans* | LUBS5 | 25.20 | 99.00% | 37.5 | I |
| *P. infestans* US-1 | *P. infestans* | P6515 | 14.85 | 98.68% | 34.4 | I |
| *P. infestans* US-1 | *P. infestans* | P8141 | 24.06 | 97.87% | 35.4 | I |
| *P. infestans* US-1 | *P. infestans* | P8844 | 16.09 | 97.72% | 35.3 | I |
| *P. ipomoeae* | *P. ipomoeae* | P300 | 19.02 | 95.25% | 36.2 | I |
| *P. ipomoeae* | *P. ipomoeae* | P301 | 15.91 | 94.24% | 36.4 | I |
| *P. ipomoeae* | *P. ipomoeae* | P302 | 17.03 | 93.58% | 36.3 | I |
| *P. ipomoeae* | *P. ipomoeae* | PIC 99167 | 34.13 | 77.30% | 33.0 | M |
| *P. mirabilis* | *P. mirabilis* | P143 | 66.04 | 95.68% | 35.9 | I |
| *P. mirabilis* | *P. mirabilis* | P144 | 40.47 | 95.41% | 32.8 | M, I |
| *P. mirabilis* | *P. mirabilis* | P153 | 8.95 | 47.27% | 36.3 | I |
| *P. mirabilis* | *P. mirabilis* | P3001 | 18.57 | 62.73% | 34.0 | I |
| *P. mirabilis* | *P. mirabilis* | P7722 | 14.78 | 93.33% | 35.5 | I |
| *P. mirabilis* | *P. mirabilis* | PIC 99114 | 46.95 | 86.78% | 34.4 | I |
| *P. phaseoli* | *P. phaseoli* | F_18 | 3.92 | 50.95% | 26.0 | M |
| *P. urerae* | *P. urerae* | PSR27 | 26.01 | 92.31% | 35.8 | I |

^a^ Note isolates *P andina* 221 and 222 were reclassified to *P. infestans* in this work.

^b^Mean coverage depth was calculated by dividing the total number of mapped bases by the genome length.

^c^ Percent mapped determined by aligning back to the *P. infestans* T30-4 reference genome.

^d^ Average base quality across all reads.

^e^Sequencing was by Illumina (I), Pac Bio (P) or Minion (M) sequencing.

**S3 Table.** **Migration rates of 1c clade *Phytophthora* species from pairwise IMa3 runs used to generate data presented in Figure 4.**

|  |  | **FROM** | | | |
| --- | --- | --- | --- | --- | --- |
|  |  | ***P. infestans*** | ***P. betacei*** | ***P. mirabilis*** | ***P. andina*** |
| **TO** | ***P. infestans*** | NA | 0.037 | 0.09 | 0.512 |
|  | ***P. betacei*** | 0.202 | NA | No estimate | No estimate |
|  | ***P. mirabilis*** | 0.063 | 0.019 | NA | 0.068 |
|  | ***P. andina*** | 0.963 | No estimate | 0.036 | NA |

**S4 Table.** Summary of t-tests comparing means for all populations and diversity statistics presented in S4-S5 Figures. Asterisk (*) indicates that means are significantly different at p<0.05.

| **Population 1** | **Population 2** | **Average Number of Pairwise Differences** | **Nucleotide Diversity** | **Number of Segregating Sites** | **Tajima's D** | **Watterson's Theta** | **Ratio of Missense to Synonymous Mutations** |
| --- | --- | --- | --- | --- | --- | --- | --- |
| All | *P. andina* | * | * | * | * | * | * |
| All | *P. betacei* | * | * | * | * | * | * |
| All | *P. infestans* | * | * | * | * | * | * |
| All | *P. mirabilis* | * | * | * | * |  | * |
| All | *P. ipomoeae* | * | * | * | * | * | * |
| *P. andina* | *P. betacei* | * | * | * | * | * |  |
| *P. andina* | *P. infestans* | * | * | * | * | * | * |
| *P. andina* | *P. mirabilis* | * | * | * | * | * |  |
| *P. andina* | *P. ipomoeae* | * | * | * | * | * | * |
| *P. betacei* | *P. infestans* | * | * | * | * | * | * |
| *P. betacei* | *P. mirabilis* | * | * | * | * | * |  |
| *P. betacei* | *P. ipomoeae* | * | * | * | * | * | * |
| *P. infestans* | *P. mirabilis* | * | * | * | * | * | * |
| *P. infestans* | *P. ipomoeae* | * | * | * | * | * | * |
| *P. infestans* Historic | All  *P. infestans* | * | * | * | * | * | * |
| *P. infestans* Mexican | *P. infestans* Historic | * | * | * |  | * | * |
| *P. infestans* Mexican | All  *P. infestans* |  | * | * | * | * | * |
| *P. infestans* Modern | *P. infestans* Mexican | * |  | * | * | * | * |
| *P. infestans* Modern | *P. infestans* Historic | * | * | * |  | * | * |
| *P. infestans* Modern | All  *P. infestans* | * | * | * | * | * |  |
| *P. infestans* South American | *P. infestans* Modern | * | * | * | * | * |  |
| *P. infestans* South American | *P. infestans* Mexican | * | * | * | * | * | * |
| *P. infestans* South American | *P. infestans* Historic | * | * | * | * | * | * |
| *P. infestans* South American | All  *P. infestans* | * | * |  | * | * | * |
| *P. infestans* US-1 | *P. infestans* South American | * | * | * | * | * |  |
| *P. infestans* US-1 | *P. infestans* Modern | * | * | * | * | * |  |
| *P. infestans* US-1 | *P. infestans* Mexican | * | * | * | * | * | * |
| *P. infestans* US-1 | *P. infestans* Historic | * | * | * | * | * | * |
| *P. infestans* US-1 | All  *P. infestans* | * | * | * | * | * |  |
| *P. mirabilis* | *P. ipomoeae* | * | * | * | * | * |  |
